# Accuracy Maximization Analysis for Sensory-Perceptual Tasks: Computational Improvements, Filter Robustness, and Coding Advantages for Scaled Additive Noise

**DOI:** 10.1371/journal.pcbi.1005281

**Published:** 2017-02-08

**Authors:** Johannes Burge, Priyank Jaini

**Affiliations:** 1 Department of Psychology, University of Pennsylvania, Philadelphia, PA, United States of America; 2 Neuroscience Graduate Group, University of Pennsylvania, Philadelphia, PA, United States of America; 3 David R. Cheriton School of Computer Science, University of Waterloo, Waterloo, ON, Canada; Northwestern University, UNITED STATES

## Abstract

Accuracy Maximization Analysis (AMA) is a recently developed Bayesian ideal observer method for task-specific dimensionality reduction. Given a training set of proximal stimuli (e.g. retinal images), a response noise model, and a cost function, AMA returns the filters (i.e. receptive fields) that extract the most useful stimulus features for estimating a user-specified latent variable from those stimuli. Here, we first contribute two technical advances that significantly reduce AMA’s compute time: we derive gradients of cost functions for which two popular estimators are appropriate, and we implement a stochastic gradient descent (AMA-SGD) routine for filter learning. Next, we show how the method can be used to simultaneously probe the impact on neural encoding of natural stimulus variability, the prior over the latent variable, noise power, and the choice of cost function. Then, we examine the geometry of AMA’s unique combination of properties that distinguish it from better-known statistical methods. Using binocular disparity estimation as a concrete test case, we develop insights that have general implications for understanding neural encoding and decoding in a broad class of fundamental sensory-perceptual tasks connected to the energy model. Specifically, we find that non-orthogonal (partially redundant) filters with scaled additive noise tend to outperform orthogonal filters with constant additive noise; non-orthogonal filters and scaled additive noise can interact to sculpt noise-induced stimulus encoding uncertainty to match task-irrelevant stimulus variability. Thus, we show that some properties of neural response thought to be biophysical nuisances can confer coding advantages to neural systems. Finally, we speculate that, if repurposed for the problem of neural systems identification, AMA may be able to overcome a fundamental limitation of standard subunit model estimation. As natural stimuli become more widely used in the study of psychophysical and neurophysiological performance, we expect that task-specific methods for feature learning like AMA will become increasingly important.

## Author Summary

In psychophysics and neurophysiology, the stimulus features that are manipulated in experiments are often selected based on intuition, trial-and-error, and historical precedence. Accuracy Maximization Analysis (AMA) is a Bayesian ideal observer method for determining the task-relevant features (i.e. filters) from natural stimuli that nervous systems *should* select for. In other words, AMA is a method for finding optimal receptive fields for specific tasks. Early results suggest that this method has the potential to be of fundamental importance to neuroscience and perception science. First, we develop AMA-SGD, a new version of AMA that significantly reduces filter-learning time, and use it to learn optimal filters for the classic task of binocular disparity estimation. Then, we find that measureable, task-relevant properties of natural stimuli are the most important determinants of the optimal filters; changes to the prior, cost function, and internal noise have little effect on the filters. Last, we demonstrate that some ubiquitous properties of neural systems, generally thought to be biophysical nuisances, can actually improve the fidelity of neural codes. In particular, we show for the first time that scaled additive noise and redundant (non-orthogonal) filters can interact to sculpt uncertainty due to internal noise to match task-irrelevant natural stimulus variability.

## Introduction

Perception science seeks to determine how perceiving organisms estimate behaviorally relevant properties of the environment based on proximal stimuli captured by the senses. Understanding the details of the sensory-perceptual processing that support these abilities with natural stimuli is a primary focus of research. It is widely appreciated that some stimulus features are more useful for some tasks than others, more likely to increase a given neuron’s response rate than others, and more likely to excite neurons in one brain area than another. This specificity suggests that perceptual and neural performance in particular tasks is driven by sets of features that are of much lower dimensionality than the proximal stimuli themselves. As a consequence, methods for reducing stimulus dimensionality are in widespread use in perception and neuroscience research.

Models of information encoding with natural stimuli are often developed without regard to what information will be decoded from the encoded signals. Efficient coding, and many statistical methods for data characterization (e.g. PCA, ICA), are designed to capture statistical properties of proximal (observable) stimuli without explicit consideration of the sensory-perceptual or behavioral goals for which the encoded information will be used [1,2] [3–9]. The efficient coding hypothesis has been remarkably influential. However, as Simoncelli & Olshausen (2001) point out, the hypothesis “states only that information must be represented efficiently; it does not say anything about what information should be represented” [7]. Empirical studies in psychophysics and systems neuroscience often focus on the behavioral limits and neurophysiological underpinnings of performance in specific tasks [10–21]. Thus, there is a partial disconnect between popular task-independent theories of encoding (e.g. efficient coding) and the methodological practices often followed by psychophysics and sensory and systems neuroscience.

Accuracy Maximization Analysis (AMA) provides a principled, data-driven approach to finding the stimulus features that are most useful for specific tasks (e.g. estimation of a variable latent in the stimulus) [22]. AMA thus addresses a need that is not directly addressed by standard efficient encoding frameworks. In conjunction with carefully calibrated natural image databases [21–26], AMA has provided predictions for the encoding filters (receptive fields) that support optimal performance in several fundamental tasks in early vision [21–24,26]. These receptive fields have, in turn, aided the development of ideal observers for the estimation of figure-ground, defocus blur, binocular disparity, retinal speed, and motion-in-depth [21,23,24,27]. The predictions of these ideal observers are biologically plausible, dovetail with available neurophysiological data, and can tightly predict human performance with natural and artificial stimuli [21]. These results may represent the beginnings of an important step forward in our ability, as a science, to develop ideal observer theories of mid-level visual tasks that act directly on natural retinal images.

AMA does not come without a set of constraints and disadvantages. The most important constraint is that the stimuli must be contrast normalized before processing. This constraint is appropriate for many perceptual tasks for which the task-relevant information is contained in the pattern of contrast over space and time, but it renders the method ill-suited for tasks in which the primary source of information is contained in the magnitude (intensity) of a stimulus. Second, the AMA cost landscape is non-convex, so guarantees cannot be made that local minima found by the method represent the global minimum; standard techniques for protecting against non-global local minima must be used (e.g. random starts). However, for the set of problems for which AMA is well-suited, its most glaring disadvantage is its computational cost: compute time is quadratic in the number of elements in the training set. Without specialized computing resources, the computational cost renders the method impractical for use on large-scale problems.

The aims of this paper are four-fold. First, to set our contribution in context, we re-derive the original equations for AMA [22], developing intuitions along the way. Second, we derive the gradient of the cost (objective function) for two popular cost functions—0,1 cost (L0 norm) and squared error cost (L2 norm)—and implement a stochastic gradient descent procedure for filter learning, which we call AMA-SGD. (source code at: http://www.github.com/BurgeLab/AMA). These advances significantly reduce the method’s compute time, thereby rendering it a more practical tool for research on problems of wide spread interest in vision research and sensory and systems neuroscience. Third, we show that AMA can be used to examine the relative impact on optimal coding of stimulus variability and priors over the latent variable. Fourth, we show how scaled additive encoding noise (i.e. additive noise with response variance proportional to the response mean) and correlated (i.e. non-orthogonal) filters can interact to confer coding advantages in certain tasks. We believe that the work presented here may help establish a general normative framework for understanding the diversity of tuning and response properties exhibited by neurophysiological receptive fields in cortex, and how they may contribute to task-specific processing of sensory stimuli.

## Methods

In this section, we first review the derivation of the main equations for Accuracy Maximization Analysis [22], explaining the logic and geometric intuitions behind the method. This review is meant to provide context for the current paper and a tutorial on the original method. Second, we derive the gradient of the cost function with respect to the filters for two popular cost functions. Third, we develop a constrained batch stochastic gradient descent algorithm for filter learning, and provide recommendations to users for best practices.

### Background and Setup

**Fig 1:**
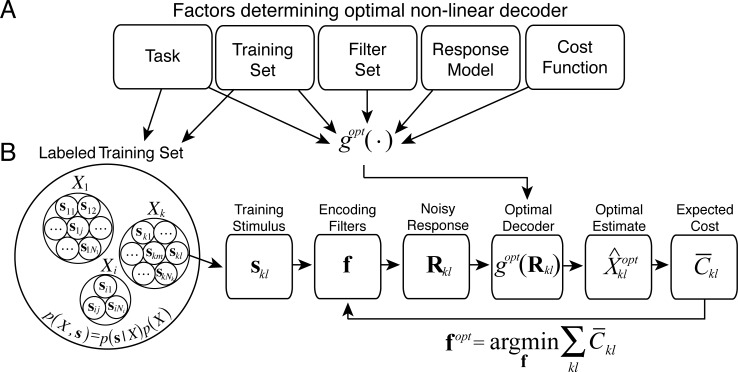
The logic of Accuracy Maximization Analysis. **A** Factors that determine the optimal non-linear decoder, *g*(⋅). For any particular filter set, the optimal decoder provides a closed form expression for the cost by i) computing the posterior probability over the latent variable *p*(*X*|**R**), and ii) reading out the optimal estimate ${\hat{X}}^{opt}$ from the posterior that minimizes the cost. **B** AMA begins with a labeled training set. Each individual stimulus in the training set, **s**_*ij*_, must be labeled with a particular value of the latent variable of interest, *X*_*i*_. The labeling of the training set implicitly defines the task. Subsequent steps to finding optimal task-specific filters via AMA are: i) select a particular stimulus **s**_*kl*_ from the labeled training set; ii) obtain a noisy filter response **R**_*kl*_ from a given (possibly non-optimal) set of initial filters; iii) use the optimal non-linear decoder to obtain the optimal estimate and its expected cost; iv) repeat for each stimulus in the training set and compute the average cost across the training set; v) update the filters to reduce the cost; vi) repeat until the average cost across the training set is minimized. The filters that minimize the cost are the optimal task-specific filters.

Accuracy Maximization Analysis (AMA) provides a closed-form expression for the optimal (nonlinear) decoding rule given five factors: i) a well-defined task (i.e. a latent variable to estimate from high-dimensional stimuli), ii) a labeled training set of stimuli, iii) a particular set of filters (receptive fields), iv) a noisy filter response model, and v) a cost function (Fig 1A). Given these factors, the problem of finding the encoding filters that are optimal for a particular task reduces to searching for the filters that minimize the cost (Fig 1B). The Background and Setup section is ordered to follow the block diagram in Fig 1A.

#### Specifying the task with a labeled training set

Accuracy Maximization Analysis requires a training set. Each stimulus in the training set is labeled by a value of the latent variable to be estimated. The task is implicitly defined by the labeling of the training set. If the training set is too small, or if the stimuli contained within the training set are not representative, results obtained via AMA may generalize poorly. The task-specific filters learned via AMA are therefore only as solid as the training set itself. Thus, the first (and often quite difficult) step in the fruitful use of AMA is to obtain labeled training sets that are accurate, and are sufficiently large to be representative of the general case.

The training set and the latent variable labels define the task and specify the joint probability distribution *p*(*X*,**s**) between the latent variable and the stimuli (Fig 1B). Thus, the training set implicitly defines the prior probability distribution over the latent variable, which can be obtained by marginalizing out the stimuli from the joint distribution: $p(X)=\sum_{s}p(X,s)$. If AMA is being used to make normative prescriptions for the design of biological and/or machine vision systems, it is of potential interest to examine the influence of the prior on the encoding functions, and on eventual performance in the task. The experimenter has at least two options in this regard.

First, the experimenter can attempt to match the prior probability distribution in the training set to the prior probability of occurrence in natural viewing conditions. Unfortunately, accurate measurements of prior probability distributions relevant to particular perceptual tasks have proved notoriously difficult to obtain, especially if the latent variable of interest is i) a property of the distal environment (e.g. depth, object motion, surface reflectance), or ii) a property of the relationship between the environment and the vision system (e.g. distance, focus error, binocular disparity, retinal image motion). Progress has been made in recent years [26,28–30], but with this approach comes significant technical challenges.

**Fig 2:**
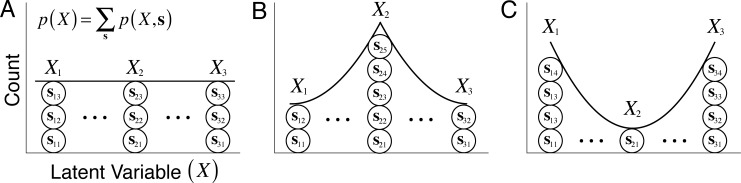
The training set implicitly represents the prior probability distribution over the latent variable to be estimated. Different prior probability distributions can be represented by varying the number of stimuli in the training set at each level of the latent variable. **A** Flat prior probability distribution over the range of represented latent variable values. **B** Prior probability with more mass at the central value of the latent variable. **C** Prior probability with less mass at the central value of the latent variable. By manipulating the number of training set stimuli as a function of the latent variable, the effect of the prior can be examined on the optimal task-specific encoding filters.

Second, the experimenter can manipulate the prior probability distribution over the latent variable by varying the number of stimuli per latent variable value in the training set. This approach is simple (in comparison to the first approach) and provides the experimenter a useful tool for examining the influence of the prior on the properties of the optimal filters Fig 2). If the optimal filters are brittle—that is, if they are very sensitive to modest variations in the shape of the prior—then the effort required by the first approach may be justified. On the other hand, if the optimal filters are insensitive to reasonable variations in the prior, then the prior can be safely ignored [24]. In general, the better the information in the proximal stimuli about the latent variable (the more reliable the measurements), the less important will become the prior.

#### Filter response model

The response model specifies how a particular filter **f** responds to an arbitrary stimulus **s**, thereby providing the conditional probability *p*(*R*|**s**) of a noisy filter response *R* to an arbitrary proximal stimulus **s** (see Fig 1B). Given that our specific interest is to understand task-specific information processing in sensory-perceptual tasks, it is advantageous for the encoding model to be consistent with the properties of biological encoders (i.e. receptive fields). Here, we consider a Gaussian response model with scaled additive (i.e. Poisson-like) noise[22]. We chose this response model for two reasons. First, its Gaussian form is mathematically convenient. Second, and more importantly, scaled additive noise is a widely accepted simple model of neural noise in early visual cortex. However, the filter response model can in principle be modified to be consistent with other noise models (e.g. Poisson, Bernoulli).

For a given encoding filter **f**_*t*_ from set of filters **f** = [**f**_1_ **f**_2_ ⋯ **f**_*q*_], its mean response *r*_*t*_, noisy response *R*_*t*_, noise samples *η*, and response noise variance $\sigma_{t}^{2}$ to stimulus *j* having latent variable level *i* are given by

$$r_{ij,t}=f_{t}^{T}s_{ij}$$ (1a)

$$R_{ij,t}=r_{ij,t}+\eta$$ (1b)

$$\eta ∼N(0,\sigma_{ij,t}^{2})$$ (1c)

$$\sigma_{ij,t}^{2}=\alpha |r_{ij,t}|+\sigma_{0}^{2}$$ (1d)

where $s_{ij}=\frac{x_{ij}-{\bar{x}}_{ij}}{\left\|x_{ij}-{\bar{x}}_{ij}\right\|}$ is a mean-subtracted, contrast normalized (‖**s**‖ = 1.0) version of a (possibly noisy) intensity stimulus **x**_*ij*_, **f**_*t*_ is a vector of encoding weights constrained to have a magnitude of 1.0 (‖**f**_*t*_‖ = 1.0), *η* is a sample of zero-mean Gaussian noise with variance $\sigma_{ij}^{2}$. In the general case, the noise variance is given by a linear function of the mean response with fano-factor *α* and baseline variance $\sigma_{0}^{2}$. When the fano-factor equals 0.0, the noise model is additive and the response variance is a constant, regardless of the mean response. When the fano-factor is non-zero, response noise variance increases approximately in proportion to the mean response. For the results presented in the paper, we set the fano-factor equal to 1.36 and the baseline variance equal to 0.23 (spk/sec)^2^, values that are consistent with neural response properties in early visual cortex [22,31,32]. If *N*_*q*_ filters are considered simultaneously, the variables in Eqs 1a–1d become vectors—mean response vector **r** = [*r*_1_ *r*_2_ ⋯ *r*_*q*_], noisy response vector **R** = [*R*_1_ *R*_2_ ⋯ *R*_*q*_], and response covariance matrix ∑ with on-diagonal elements $diag\left(\Sigma \right)=\left(\sigma_{1}^{2},\sigma_{2}^{2},\cdots ,\sigma_{q}^{2}\right)$—and the filter response distribution *p*(**R**|**s**_*ij*_) becomes *N*_*q*_ dimensional. In this manuscript, we consider independent response noise (diagonal covariance matrix), but the impact of correlated response noise could also be examined.

#### Bayes Optimal Decoder: Posterior Probability Distribution and Cost of Optimal Estimator

The optimal decoder provides a closed form expression for the cost for any particular filter set given the training stimuli. The decoder determines the cost by first computing the posterior probability over the latent variable *p*(*X*|**R**), and then reading out the optimal estimate ${\hat{X}}^{opt}$ from the posterior that minimizes the cost. Here, following Geisler et al (2009), we present the derivation of the posterior probability of the latent variable *X* in a labeled training set given the responses of a noisy set of encoders (i.e. filters) to a given stimulus **s**_*kl*_ with latent variable value *X*_*k*_

$$p(X_{k}|R(k,l))=\frac{p(R(k,l)|X_{k})p(X_{k})}{\sum_{i=1}^{N_{lvl}}p(R(k,l)|X_{i})p(X_{i})}$$ (2)

The conditional probability of the encoder response given can be expressed as $p\left(R|X_{i}\right)=\sum_{i=1}^{N_{i}}p\left(R|s_{ij}\right)p\left(s_{ij}|X_{i}\right)$ where *p*(**R**|**s**) is defined by Eqs 1a–1d. Plugging in

$$p(X_{k}|R(k,l))=\frac{\left[\sum_{m=1}^{N_{k}}p(R(k,l)|s_{km})p(s_{km}|X_{k})]p(X_{k}\right)}{\sum_{i=1}^{N_{lvl}}\left[\sum_{j=1}^{N_{i}}p(R(k,l)|s_{ij})p(s_{ij}|X_{i})]p(X_{i}\right)}$$ (3)

Next, note the prior probability *p*(*X*_*i*_) is known, and the conditional probability of a particular stimulus given a level *p*(**s**_*ij*_|*X*_*i*_) is also known (because these quantities are determined by the training set). Specifically, the prior probability of each latent variable value *p*(*X*_*i*_) is the number of stimuli having that label over the total number of stimuli in the training set *N*_*i*_/*N*. The probability of each stimulus, conditioned on its latent variable value *X*_*i*_ is 1/*N*_*i*_ where *N*_*i*_ is the number of stimuli with that label in the training set. Substituting

$$p(X_{k}|R(k,l))=\frac{[\sum_{m=1}^{N_{k}}p(R(k,l)|s_{km})\frac{1}{N_{k}}]\frac{N_{k}}{N}}{\sum_{i=1}^{N_{lvl}}[\sum_{j=1}^{N_{i}}p(R(k,l)|s_{ij})\frac{1}{N_{i}}]\frac{N_{i}}{N}}$$ (4)

Canceling terms yields the relatively simple expression for the posterior probability

$$p(X_{k}|R(k,l))=\frac{\sum_{m=1}^{N_{k}}p(R(k,l)|s_{km})}{\sum_{i=1}^{N_{lvl}}\sum_{j=1}^{N_{i}}p(R(k,l)|s_{ij})}$$ (5)

**Fig 3:**
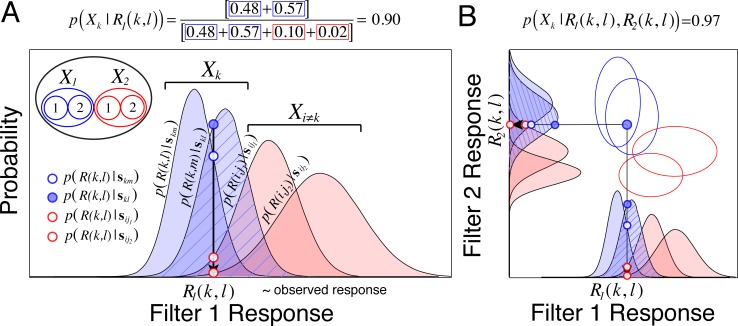
Relationship between filter response distributions, the likelihood, and the posterior probability. **A** Hypothetical one-dimensional conditional response distributions from a single filter (receptive field). Each distribution represents noisy filter responses to each stimulus in the training set. Blue distributions represent the filter response distributions for the two stimuli having the first latent variable value. Red distributions respresent the response distributions to the two stimuli having the second (i.e. incorrect) value. The striped blue distribution corresponds to the distribution of responses to the current stimulus **s**_*kl*_ which, in this case, has the first value of the latent variable. The solid blue circle represents the likelihood that a random observed response *R*_1_(*k*,*l*) was elicited by stimulus **s**_*kl*_ the stimulus that actually elicited the response. The open blue circle represents the likelihood that the same response was elicited by stimulus **s**_*km*_, the other stimulus having latent value *X*_*k*_. The sum of these stimulus likelihoods represents the likelihood that the observed response was elicited by a stimulus having latent variable value, *X*_*k*_. The open red circles represent the likelihoods that the observed response resulted from the two stimuli having value *X*_*i* ≠ *k*_ (i.e. from stimuli with the incorrect latent variable value). The posterior probability (Eq 5) of the correct latent variable value (i.e. the latent variable value *X*_*k*_ corresponding to stimulus **s**_*kl*_) is given by the sum of the likelihoods for within-level stimuli normalized by the sum of all likelihoods; the posterior probability distribution for this hypothetical case is shown in the next figure. Confusions between stimuli with the correct latent variable value increase the posterior probability of the correct level by contributing to the numerator (blue-boxed entries in the equation above figure panel). Confusions between correct and incorrect levels decrease the posterior probability of the correct level by contributing to the denominator (red-boxed entries in equation above figure panel). **B** Two-dimensional (i.e. two filter) case, under the assumption of independent response noise (note how the noise ellipses are aligned with the axes of response space). The second filter should help increase performance by selecting for useful stimulus features that the first filter does not.

Eq 5 indicates that the posterior probability is given by the sum of the within-level stimulus likelihoods, normalized by the sum of all stimulus likelihoods. Fig 3A provides a graphical representation of AMA posterior, for a simple hypothetical case in which there is one filter and two latent variable values, each with two stimuli (i.e. four stimuli total). Fig 3B shows response distributions for the same hypothetical stimuli, in the slightly more complicated case in which there are two filters.

With the expression for the posterior probability distribution, the next step is to define a cost function. The cost function specifies the penalty assigned to different types of error. For certain cost functions, the optimal estimator associated with that cost function can be determined analytically (see Supporting Information). Here, we remain agnostic about the particular cost function to be used. Later, we derive the cost (and the gradient of the cost) associated with two popular cost functions for which the maximum a posteriori (MAP) and minimum measured squared error estimators (MMSE) are the optimal estimators.

The cost associated with the noisy response to an individual stimulus is

$$C_{kl}=\sum_{u=1}^{N_{lvl}}\gamma \left({\hat{X}}^{opt},X_{u}\right)p\left(X_{u}|R\left(k,l\right)\right)$$ (6)

where $\gamma \left({\hat{X}}^{opt},X_{k}\right)$ is the cost associated with the difference between the estimate and the true latent variable value *X*_*k*_ when the estimate is the optimal estimate ${\hat{X}}^{opt}$ for the cost function.

The overall cost for a given set of filters applied to the training set data is given by the expected cost across for each stimulus averaged over all stimuli

$$\begin{array}{l} \bar{C}=\frac{1}{N}\sum_{k,l}^{N}E_{R(k,l)}[C_{kl}]\\ \;=\frac{1}{N}\sum_{k,l}^{N}{\bar{C}}_{kl} \end{array}$$ (7)

where ${\bar{C}}_{kl}=E_{R\left(k,l\right)}\left[C_{kl}\right]$ is the expected cost associated with the *kl*^th^ stimulus.

The goal of the accuracy maximization analysis is to obtain the filters **f** that minimize the overall cost. Namely,

$$f^{opt}=\underset{f}{\arg\;\min}\;\bar{C}$$ (8)

where the optimal filters **f**^*opt*^ are the filters that minimize the expected cost across the training set. We use numerical methods to determine the optimal filters because there exists no closed form solution.

**Fig 4:**
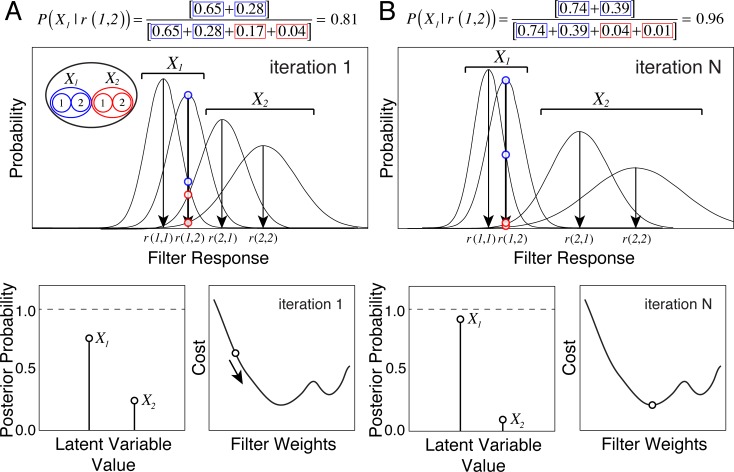
Schematic showing the evolution of hypothetical filter response distributions, posterior probability distributions, and cost with one filter, two latent variable levels, and two stimuli per level. **A** Response distributions, posterior, and cost for intermediate filters. **B** Response distributions, posterior, and cost for optimal filters. Three effects are worth noting. First, as the filters evolve, response distributions to stimuli sharing the same latent variable value become more similar, and response distributions to stimuli having different latent variable values become more different. Second, as the filters improve, more posterior probability mass is concentrated at the correct latent variable value, and cost decreases commensurately. Third, the cost landscape is non-convex.

A schematic of the filter learning process via gradient descent is shown in Fig 4. It shows how the filter response distributions, the corresponding posterior probability distribution over the latent variable, and the cost evolves as the filters improve. As the filters improve, response distributions to stimuli having the same level of the latent variable become more similar, while response distributions to stimuli with different latent variable values become more dissimilar. This increases the likelihood of within-level stimulus confusions, and decreases the likelihood of between-level stimulus confusions.

### AMA Cost Function: Derivation of Gradients

Gradient descent routines require the gradient of the cost function. The gradient must be determined numerically (e.g. finite differences) if an analytic expression is not known. The computational cost of numerically evaluating the gradient is proportional to the number of dimensions required to define the gradient itself. Methods for numerical evaluation typically proceed by taking a small step of size *ε* in each of *N*_*d*_ directions. In our case, *N*_*d*_ is the number of dimensions that define each filter (i.e. receptive field). For example, a filter that processes a 15x15 pixel image is defined by 225 dimensions, one dimension for each pixel. Thus, the gradient of the cost with respect to the filter is 225 dimensional. An analytical expression for the gradient can be expected to yield computational savings equal to a factor *N*_*d*_ less the time required to evaluate the gradient. This improvement in speed can be substantial for problems in which the stimuli and filters are relatively high dimensional.

Here, we derive the gradient of the cost for two popular cost functions: the 0,1 cost function (i.e. L0 norm) and the squared error cost function (i.e. L2 norm). These two cost functions are commonly used in the fields of vision research, visual neuroscience, statistics, and machine learning. They also represent opposite extremes of commonly used cost functions. The 0,1 cost function penalizes all errors equally, regardless of their magnitude. The squared error cost function penalizes small errors minimally and large errors severely. We reason that if the behavior of the algorithm is understood for these two cost functions, reasonable inferences can be made about algorithm’s behavior for intermediate cost functions (e.g. L1 norm).

The optimal estimator for the L0 norm cost function is the maximum a posteriori (MAP) estimator (see S4 Text). In the present case, the expected L0 cost across all stimuli is closely related to the Kullback-Leibler (KL) divergence between the observed posterior and an idealized posterior with all its mass at the correct level of the latent variable (S5 Text); for both statistics, the expected cost is a function only of the probability mass at the correct level of the latent variable. Thus, the appropriate estimator for both measures is the posterior maximum (i.e. the MAP estimator). The optimal estimator for the L2 norm cost function is the mean of the posterior probability distribution, or the minimum mean squared error (MMSE) estimator (see S6 Text).

#### Gradient of 0,1 (L0 norm) cost function

This expression for the 0,1 cost is closely related to the average KL-divergence between the posterior probability distribution and a hypothetical posterior probability distribution that has all its mass at the correct latent variable level (S5 Text). The KL-divergence cost for a noisy response to a particular stimulus is given by the negative log-posterior probability at the correct level [22]

$$C_{kl}=-\log\;p\left(X_{k}|R\left(k,l\right)\right)$$ (9)

The expected cost across all stimuli depends on the expected cost for each individual stimulus (Eq 7). We use the approximation log *p*(*X*_*k*_|**r**(*k*,*l*)) ≅ *E*_**R**(*k*,*l*)_[log *p*(*X*_*k*_|**R**(*k*,*l*))] to calculate the expected cost for each stimulus (see Appendix, [22]). Note that AMA-SGD can learn filters with noisy responses and without the approximation, but results are robust to this choice, so we use the approximation for convenience.

By defining *Y*_*k*_(*k*,*l*) and *Z*(*k*,*l*) as the numerator and denominator of the posterior probability distribution given a noisy response **R**(*k*,*l*) to stimulus **s**_*kl*_ (see Eq 5), we obtain

$$C_{kl}=-\left[\log Y_{k}\left(k,l\right)-\log Z\left(k,l\right)\right]$$ (10)

Taking the gradient of the cost with respect to the receptive fields **f** and dropping the index (*k*,*l*) for notational simplicity yields

$$\nabla_{f_{q}}C_{kl}=-\left[\frac{\nabla_{f_{q}}Y_{k}}{Y_{k}}-\frac{\nabla_{f_{q}}Z}{Z}\right]$$ (11)

Finally, plugging Eq 11 into Eq 7, and yields the expression for the expected cost over the entire training set

$$\nabla_{f_{q}}\bar{C}=-\frac{1}{N}\sum_{kl}^{N}\left[\frac{\nabla_{f_{q}}Y_{k}}{Y_{k}}-\frac{\nabla_{f_{q}}Z}{Z}\right]$$ (12)

Thus, finding the gradient of the KL-divergence cost reduces to finding the gradient of the log posterior probability, which further reduces to finding the gradient of the numerator and the gradient of the denominator of the posterior probability distribution. S2 Text derives the full expression for the gradient of the cost. The accuracy of the analytic expressions for the gradient have been verified by numerical evaluation using finite differences.

#### Gradient of squared error (L2 norm) cost function

The squared error between the groundtruth value of the latent variable and the optimal estimate given a noisy response to a particular stimulus is

$$C_{kl}={\left({\hat{X}}_{kl}^{opt}-X_{k}\right)}^{2}$$ (13)

The gradient of the cost is

$$\nabla_{f_{q}}C_{kl}=2\left({\hat{X}}_{kl}^{opt}-X_{k}\right)\nabla_{f_{q}}{\hat{X}}_{kl}^{opt}$$ (14)

The optimal estimate for the squared error (i.e. L2 norm) cost function is the posterior mean (see S4 Text). The gradient of the optimal estimate is given by

$$\nabla_{f_{q}}{\hat{X}}_{kl}^{opt}=\sum_{u=1}^{N_{lvl}}X_{u}\left[\nabla_{f_{q}}p\left(X_{u}|R\left(k,l\right)\right)\right]$$ (15)

The gradient of the posterior probability (S3 Text) at each level of the latent variable is given by

$$\nabla_{f_{q}}p\left(X_{u}|R\left(k,l\right)\right)=\frac{Y_{u}}{Z}\left(\frac{\nabla_{f_{q}}Y_{u}}{Y_{u}}-\frac{\nabla_{f_{q}}Z}{Z}\right)$$ (16)

where *Y*_*u*_ and *Z* are the numerator and denominator of the posterior probability, as above. By substituting Eq 16 into Eq 15, we obtain the gradient of the optimal estimate

$$\nabla_{f_{q}}{\hat{X}}_{kl}^{opt}=\sum_{u=1}^{N_{lvl}}X_{u}\left[\frac{Y_{u}}{Z}\left(\frac{\nabla_{f_{q}}Y_{u}}{Y_{u}}-\frac{\nabla_{f_{q}}Z}{Z}\right)\right]$$ (17)

Substituting Eq 17 into Eq 14, using an approximation (see Appendix, [22]), substituting into Eq 7, and taking the gradient yields the expression for the gradient of cost over the training set

$$\nabla_{f_{q}}\bar{C}=\frac{2}{N}\sum_{kl}^{N}\left({\hat{X}}_{kl}^{opt}-X_{k}\right)\nabla_{f_{q}}{\hat{X}}_{kl}^{opt}$$ (18)

The full derivation for the gradient of the squared error cost is given in S3 Text. With the gradient of the cost in hand, we develop a stochastic gradient descent routine for finding the optimal filters. The accuracy of the analytic expressions for the gradient have been verified by numerical evaluation of the gradient using finite differences.

### AMA-SGD (Accuracy Maximization Analysis with Stochastic Gradient Descent)

#### Motivation: reducing computational run-time

The primary drawback of AMA is its computational expense. The compute time associated with the evaluation of the posterior probability distribution for all stimuli in the dataset requires *N*^2^*N*_*lvl*_ operations, where *N* is the total number of samples in the training set and *N*_*lvl*_ is the number of levels (i.e. values) of the latent variable represented in the training set. For example, a training set with 10,000 stimuli and 20 categories requires 2 billion operations per evaluation of the posterior probability distribution. The required compute time is significant enough as to render the method impractical for use on large-scale problems.

There are at least two methods for achieving significant computational savings in optimization problems: employing stochastic gradient descent routines, and employing models with strong distributional or parametric assumptions. Each has its drawbacks. Stochastic gradient descent routines are noisy and may not converge to the optimum value when the problem is non-convex. Models with strong parametric assumptions will, in general, only be appropriate for a restricted set of cases for which the assumptions approximately hold. Both approaches, however, offer the potential benefit of drastic improvements in the speed of convergence. In this paper, we focus on stochastic gradient descent; future work will explore models with stronger parametric assumptions.

Stochastic gradient descent has the potential to significantly reduce compute-time when the time to evaluate the objective function increases super-linearly with the number of elements in the training set [33–35], which is the case here. The expected reduction in compute-time depends on the size of the batch relative to the size of the full training set. On each iteration, a batch of stimuli of size *N*_*bch*_ is selected randomly from the total number of stimuli in the training set. Let *k* = *N*/*N*_*bch*_ be the ratio between the size of the training set and the size of each batch. Evaluating the *N*_*bch*_ posterior probability distributions associated with each batch requires $\frac{N^{2}}{k^{2}}N_{lvl}$ operations. On each pass through the dataset, *k* batches must be evaluated so that the full training set is used during filter learning. All other things equal, evaluating the cost for each pass through the full dataset is therefore of order *N*_*bch*_*N*_*lvl*_*N*, a factor of *k* faster than AMA. Thus, AMA-SGD has the potential to reduce the time required to learn filters from quadratic to linear in the number of elements in the training set.

#### Updating the filters

The problem under consideration is a constrained optimization problem because the filters must have a vector magnitude (L2 norm) of 1.0. The geometric interpretation of this constraint is that the filters lie on a hyper-sphere of unit radius that is centered at the origin. Therefore, the direction of steepest descent that satisfies the constraint lies on the tangent plane of the hyper-sphere at the point specified by the current filter values **f**.

**Fig 5:**
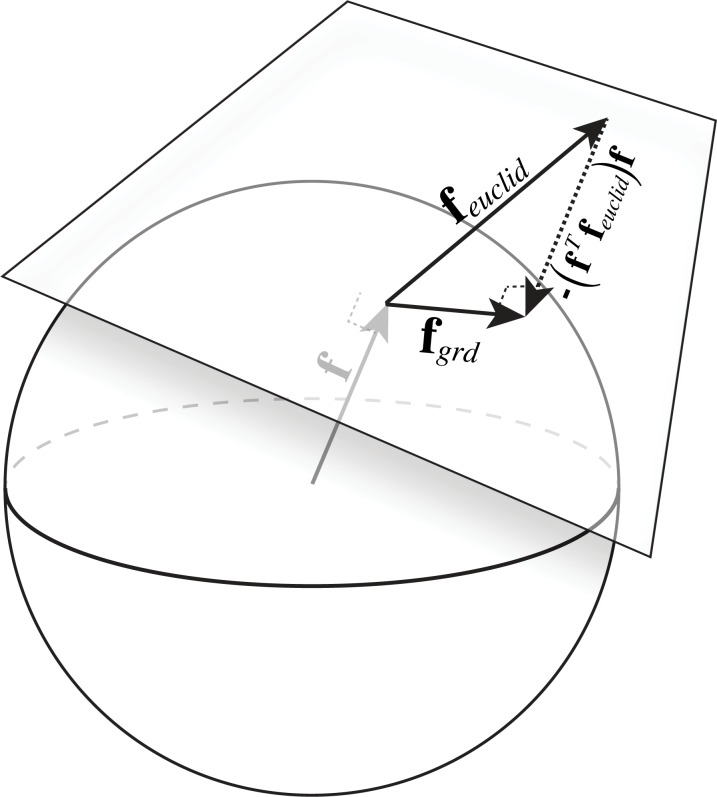
Using projection to satisfy the filter constraint. **A** To enforce the constraint that the filters have unit vector magnitude (i.e. ‖**f**‖ = 1.0), the filters are projected onto the tangent plane of the unit hypersphere. The vector difference between the gradient in the unconstrained space **f**_*euclid*_, and the projection of that gradient onto a unit vector perpendicular to the tangent plane of the hypersphere at **f** (which is identically equal to **f**) gives the gradient of the cost in the tangent plane of the hypersphere **f**_*grd*_. Changing the value of the filters by taking a small step in the direction of **f**_*grd*_ ensures that the step will be in the direction that reduces the cost the fastest while still satisfying the constraint that the vector magnitude (i.e. L2 norm) of the filter remain 1.0.

To determine this direction, the gradient of the cost function in the unconstrained space is first obtained, **f**_*euclid*_ (Eqs 12 and 18). Next, the gradient in the unconstrained space is projected onto the tangent plane of the hypersphere (Fig 5). The gradient in the unconstrained space can be expressed as a vector sum of its component in the tangent plane and its component in a direction perpendicular to the tangent plane at the point **f**. On a hyper-sphere, the direction perpendicular to the tangent plane at **f** is the vector **f** itself. Hence, the projection of the gradient in this direction is (**f**^*T*^**f**_*euclid*_)**f**. Therefore, from vector addition, the projection of the gradient on the tangent plane of the sphere at **f** is

$$f_{grd}=f_{euclid}-(f^{T}f_{euclid})f$$ (19)

The unit vector **f**_*grd*_/‖**f**_*grd*_‖ therefore represents the direction satisfying the constraints in which the cost function is changing most rapidly.

#### Taking a step

The stochastic gradient descent algorithm is iterative. After each iteration, the filter values are updated by taking a step in the direction of steepest descent that satisfies the constraints. We take a step from the current (old) value of the receptive fields **f**^(*old*)^ to the new value of the receptive fields **f**^(*new*)^ in the direction of steepest descent that satisfies the constraint. In particular, the updated receptive fields are given by $f^{\left(new\right)}=f^{\left(old\right)}-\varepsilon \frac{f_{grd}^{\left(old\right)}}{\left\|f_{grd}^{\left(old\right)}\right\|}$ where *ε* is the step size.

A step in the direction of steepest descent generally updates the filter values such that cost decreases. However, because batch stochastic gradient descent randomly selects random batches of training stimuli on each iteration, stimuli in some batches may be ‘easy’ while stimuli in other batches may be ‘hard’. Thus, some batches may produce lower costs irrespective of the properties of the filters. Therefore, on each iteration, the updated filter values are preserved for the next iteration only if the value of the cost function for the current batch decreases after the update. By randomly choosing the batches over a large number of iterations, the algorithm, in expectation, converges to the optimum.

#### Choosing a step size

The problem of choosing an appropriate step size in a gradient descent algorithm has received a good deal of attention in the statistics and machine learning literature. Various methods have been proposed for how to choose step sizes that optimize the rate of convergence [36,37]. Many of these methods adapt the step size to the structure of the cost function, and have demonstrated desirable convergence properties. A formal investigation of how best to choose the step size is beyond the scope of this paper. We followed three basic principles. First, the step size should not be too big; otherwise the algorithm may never converge to the optimum value. Second, the step size should not be too small; otherwise, the algorithm may require a very large number of iterations to achieve convergence. Third, the step size should decrease as the number of iterations increases. We obtained good performance by programming our routine to decrease step size 1.0% on each iteration and to quit after a certain limiting number of iterations. There is clearly room for improvement in this procedure. The results presented here thus represent a lower bound on performance.

#### Choosing a batch size

The AMA-SGD method developed here uses stochastic batch gradient descent. On each iteration of a batch gradient descent method, a batch of stimuli of a certain size is chosen at random from the training set, the cost and gradient is computed from the batch, and then a step is taken in the direction of the gradient. The choice of batch size is left to the user. It is tempting to choose the smallest possible batch size because the smaller the batches, the more significant the improvement in speed (see above). However, if batch size is too small, filters learned via AMA-SGD will not converge to the filters learned with AMA (see Results). Choosing a batch size is therefore a trade-off between computational speed and accuracy.

## Results

To demonstrate the value of AMA-SGD, we use the task of estimating binocular disparity from natural stereo-images [24] as a concrete test case. In the context of this task, we show that AMA-SGD converges, dramatically improves the speed of filter learning, and returns the same filters as AMA given sufficiently large batch sizes. Then, we demonstrate that the optimal filters are highly robust to changes in the prior probability distribution, overall noise power, and cost function. We note that these results are not unique to the task of disparity estimation; similar convergence and filter robustness results are obtained for several other tasks. (Labeled training sets for the related tasks of estimating binocular disparity and retinal speed from natural stimuli are available at http://www.github.com/burgelab/AMA). Finally, in the discussion section, we examine the general implications of the these results for understanding neural coding with biologically realistic noise models (i.e. noise variance that increases with the mean).

### Binocular Disparity Estimation

**Fig 6:**
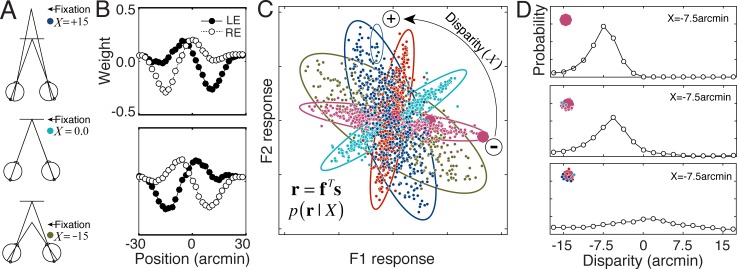
AMA results for disparity estimation with natural stereo images. **A** Stereo-geometry for three different disparities: uncrossed disparity (*δ* = -15 arcmin, eyes fixated in front of target; brown), zero disparity (*δ* = 0.0 arcmin, eyes fixated on target; turquoise), crossed disparity (*δ* = +15 arcmin, eyes fixated behind target; blue). **B** Optimal AMA filters. **C** Conditional response distributions *p*(**r**|*x*) for five different values of the disparity (i.e. latent variable): -15.0, -7.5, 0.0, +7.5, +15.0 arcmin. Each dot represents the expected joint response to an individual stereo image. The responses are the projection of the stimuli basis elements defined by the filters. The conditional response distributions are well characterized by Gaussians (large colored ellipses). For reference, a small ellipse representing filter response noise associated with one stimulus is shown (upper middle part of plot). **D** Posterior probability distributions for three stimuli having -7.5 arcmin of disparity (oversized response dots in C). The posteriors decrease in peakiness and increase in bias as the responses approach the origin, reflecting the fact that responses nearer the origin are more difficult to decode.

Binocular disparities are the local differences between the left and right eye retinal images due to the different vantage point each eye has on the world. Binocular disparities are used for fixating the eyes and for computing the depth structure of scenes (Fig 6A). But the disparities themselves must be estimated before they can be used for depth perception.

The estimation of binocular disparity is a classic problem in vision science, and is often referred to as the stereo-correspondence problem. The behavioral limits and neural mechanisms of disparity estimation have been extensively investigated [15,18,20,38–41]. However, until recently there was no ideal observer for estimating disparity in natural images [24]. To develop this ideal observer, Burge & Geisler (2014) first obtained a labeled training set of randomly selected 1 deg binocular retinal images of natural scenes with disparities ranging between -15 to 15 arcmin (400 binocular stimuli x 19 disparity levels = 7600 total). Physiological optics, and the wavelength sensitivity and spatial sampling of the foveal photoreceptors were accurately modeled. AMA was then used to find the small population (n = 8) of binocular filters that extract the most useful information in natural images for the task. Additional filters yielded little improvement, suggesting that eight binocular filters capture most of the available task-relevant information. The properties of the filters mimic the receptive fields of disparity sensitive neurons in cortex, and optimal disparity decoding predicts many aspects of human disparity estimation and discrimination performance. Please see Burge & Geisler (2014) for extensive details on the training set, the ideal observer for disparity estimation, and the role AMA played in its development.

The two most useful filters in the disparity estimation task are shown in Fig 6B. These receptive fields took approximately 1 hour to learn on a 2012 MacBook Pro. The disparity-conditioned filter responses *p*(**r**|*X*) to the contrast normalized stimuli are approximately Gaussian (Fig 6C), and the optimal filters are somewhat anti-correlated: $\rho =f_{1}^{T}f_{2}=-0.22$. Posterior probability distributions for three joint filter responses (oversized dots) are shown in Fig 6D. As the responses get farther from the origin, the posterior probability distributions have more of their mass at the correct level of the latent variable.

The filter response distributions in Fig 6C, and the manner in which they change with the value of the latent variable, are similar to the response distributions obtained for other tasks/coding problems that have been modeled with ‘energy-like’ computations (e.g. disparity-energy, motion-energy) [15,21,24,42]: the information about the latent variable is carried primarily by the covariance of the filter responses. This characteristic pattern of filter response will inform subsequent analyses of how interactions between filter correlation, response noise, and stimulus distributions impact encoding fidelity (see Discussion).

### AMA-SGD Performance

#### Convergence & run-time improvements

**Fig 7:**
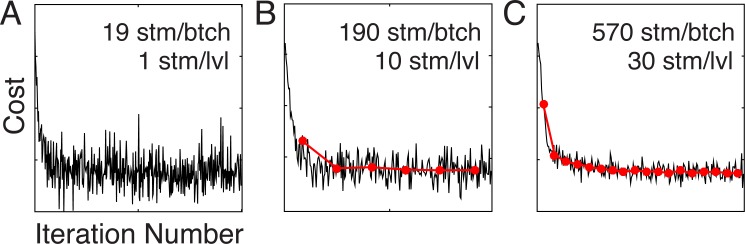
Stochastic gradient descent of cost landscape, over two hundred iterations, for three different batch sizes. Cost computed from each batch as a function of the iteration number as the filters evolve. Jagged black curves show the cost associated with each batch. Red curves show the average cost for each pass through the entire training set. More passes are made through the training set with larger batch sizes and the same number of iterations. **A** 19 stimuli per batch, one stimulus per level. **B** 190 stimuli per batch, ten stimuli per level. **C** 570 stimuli per batch, thirty stimuli per level.

In this section, we demonstrate AMA-SGD’s convergence properties. The disparity filters (c.f. Fig 6) were learned with the original AMA model and therefore constitute a benchmark for AMA-SGD. Here, we examine the effect of batch size on the convergence properties, run-time improvements, and the validity of AMA-SGD filters. Stochastic gradient descent is a noisy process by design. Thus, it is important to verify that AMA-SGD converges. Descent of the cost function should be noisier with small batches and smoother with large batches. Fig 7 confirms these expectations and shows that the cost converges noisily but systematically for a wide range of different batch sizes.

**Fig 8:**
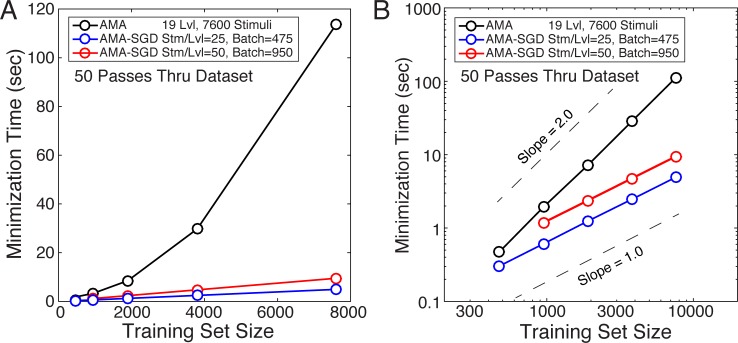
Run-time comparison between AMA and AMA-SGD. Minimization time as a function of training set size on: **A** linear-linear axes, and **B** log-log axes. Minimization time for AMA increases quadratically while AMA-SGD increases linearly (for a fixed batch size) with the number of elements in the training set. A comparison of AMA and AMA-SGD costs is shown in Fig 9.

To verify the expected improvements in run-time, we compared the time required to evaluate the cost using AMA-SGD for different batch sizes and training set sizes. Evaluating the cost with AMA-SGD is expected to be linear in the number of elements in the training set, for a fixed batch size (see Methods). Fig 8 shows the time required to evaluate the cost for 50 passes through training sets of varying size using AMA (black) and AMA-SGD with batch sizes of 475 stimuli (25 stm/lvl; red) and 950 stimuli (50 stm/lvl; blue). Results show that AMA is quadratic in the number of elements in the training set. Results also show, as expected, that the stochastic gradient descent routine is linear in the number of elements in the training set for a fixed batch size. Thus, AMA-SGD can yield dramatic improvements in the speed of filter learning.

#### Batch size effects

The faster convergence times obtained with AMA-SGD are advantageous only if the resulting filters are the same as those obtained by AMA. The previous section showed that the most dramatic reductions in run-time occur with the smallest batches. It is therefore tempting to use very small batches when learning filters. However, there is a trade-off between computational gains and accuracy of the filters. AMA-SGD only returns filters identical to those returned by AMA if the batch size is sufficiently large.

To understand why the filters critically depend on batch size, consider the case in which the batch size is so small that there is only one stimulus per level in the average batch. In this case, the probability of response conditioned on a particular value of the latent variable is identical to the probability of the response conditioned on the stimulus having that level: *p*(**R**|*X*_*i*_) = *p*(**R**|**s**_*ij*_). Thus, the posterior probability of the latent variable is identical to the posterior probability of the stimulus, and the filters that best identify the latent variable are identical to the filters that best identify each stimulus. Therefore, as the number of stimuli per level decreases to one, the distinction between identifying the latent variable and identifying a particular stimulus ceases to exist. Hence, a primary distinction vanishes between AMA and other more widely known methods for dimensionality reduction. Under these conditions, one should obtain AMA-SGD filters that are similar to PCA filters.

**Fig 9:**
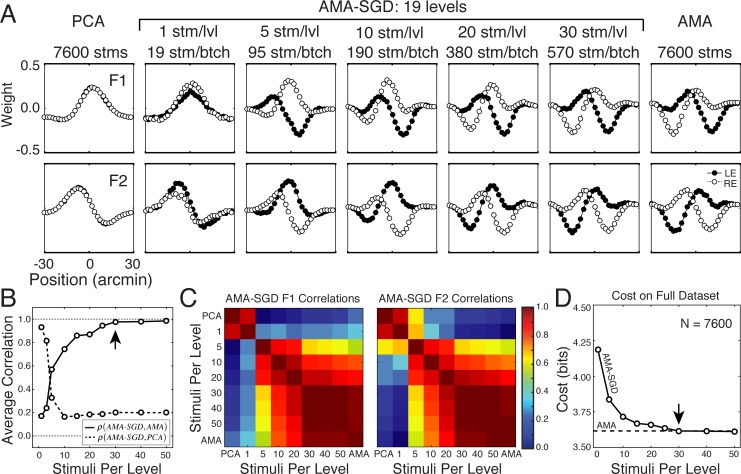
The effect of batch size on AMA-SGD filter shapes. **A** As batch size decreases to ~1 stimulus per level, AMA-SGD filters become similar to PCA filters. As batch size increases to ~30 stimuli per level, AMA-SGD filters converge to AMA filters. **B** Average AMA-SGD filter correlation with PCA filters (dashed) and AMA filters (solid) as a function of the number of stimuli per level. Arrow marks the critical number of stimuli per level, above which AMA-SGD filters are consistent with AMA filters. **C** Filter 1 and filter 2 correlation matrices. **D** Cost, computed over the full dataset with AMA-SGD filters, as a function of the number stimuli per level. Arrow marks the number of stimuli per level above which the total cost computed on the full dataset, is minimized. When learning filters via AMA-SGD, it is critical to have a sufficient number of stimuli per level.

To illustrate this point, we learned filters multiple times using AMA-SGD where the only difference between each run was the batch size (Fig 9). Indeed, we find that when the batch has only one stimulus per level (~19 stimuli/batch), the resultant AMA-SGD filters are highly correlated with PCA filters. When the batch has 30 or more stimuli per level (~570 stimuli/batch), the resultant AMA-SGD filters are highly correlated with the AMA filters that were learned using AMA (Fig 9A–9C). Costs associated with AMA and AMA-SGD filters become identical as well (Fig 9D). Thus, users should be wary of using small batch sizes when learning filters via AMA-SGD. (See S3 Fig for more on the distinction between AMA, PCA, and ICA).

We have not fully explored how many stimuli per level are required in a batch for AMA-SGD to converge to the filters returned by AMA. It most likely depends on the use case. However, for the tasks we have examined, a good rule of thumb is to start with batches having approximately 30 stimuli per latent variable level and to systematically increase the batch size until the learned filters are stable.

### Filter Robustness

In this section, we examine the robustness of the optimal filters to changes in the prior probability distribution, overall noise power, and cost function. We find that the optimal filters are remarkably stable, suggesting that natural stimulus properties are the primary determinants of the optimal filter shapes.

#### The effect of the prior

**Fig 10:**
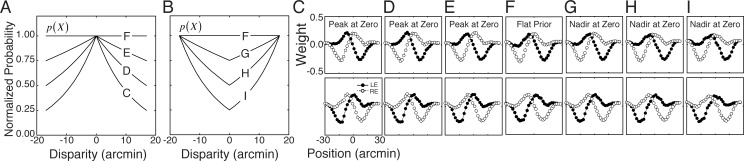
Effect of prior on optimal filters for disparity estimation. **A** Prior probability distributions used to learn the filters presented in C-E. **B** Prior probability distributions used to learn filters in G-I. **C-E** Filters obtained with prior probability distributions having peaks at zero. **F** Filters obtained with flat prior probability distribution; this prior was used throughout the main section of the paper. **G-I.** Filters obtained with prior probability distributions having less mass at zero.

In a closed system, the prior probability distribution can be experimentally manipulated, and its effects can be empirically determined. Here, we examine how the prior impacts the optimal AMA filters for the task of estimating binocular disparity with natural stimuli. The effects of seven different prior distributions are examined. The first is the flat prior probability distribution in the training set used throughout the paper: 400 natural stimuli at each of nineteen disparity levels from -15 to 15 arcmin [24]. Of the remaining six priors, three had excess probability mass at zero (zero-disparity priors; Fig 10A), and three had excess mass at large non-zero disparities(Fig 10B). These priors are enforced by randomly culling stimuli in appropriate numbers from each level of the latent variable in the training set (Fig 10A and 10B).

In general, the filter shapes are largely robust to substantial variation in the prior probability distribution. The correlations between the filters in F (flat prior) and the filters in C-I are 0.96, 0.98, 0.98, 1.00, 0.97, 0.96, and 0.96, respectively. The priors do have some effect, however. Zero-disparity priors (Fig 10A) cause the optimal filters to select for somewhat higher spatial frequencies and smaller phase shifts than a flat prior. Priors with excess mass at large non-zero disparities (Fig 10B) cause the optimal filters to select for somewhat lower spatial frequencies and larger phase shifts. These trends are as expected [24]. However, the optimal filters in the disparity estimation task are largely robust to the variations in the prior examined here. These results suggest, consistent with intuition, that the task-relevant features of natural (proximal) stimuli are the primary determinants of the optimal stimulus encoders. This result should not come as a surprise. In Bayesian signal detection theory, for example, the primary effect of a prior is to shift the decision boundary [43].

This general approach- manipulation of the prior in a closed system- may prove useful for investigations of optimal information processing in other sensory-perceptual tasks. It may also prove useful in evaluating claims in the literature about the constraints priors place on the design of neural systems and the subsequent limits of sensory-perceptual processing[44,45] in the presence of natural stimulus variation.

#### The effect of noise power

Here, we examine the effect of the overall level of encoding noise power on the optimal receptive field shapes. We considered five noise variances over a range spanning two orders of magnitude. The low noise condition contained 1/10th the original noise variance (*α* = 0.136; $\sigma_{0}^{2}=0.023$), and the high noise variance condition contained 10x the original noise variance ((*α* = 13.6; $\sigma_{0}^{2}=2.30$). To isolate the effect of cut noise variance, the training set and all other parameters were held constant across the conditions.

**Fig 11:**
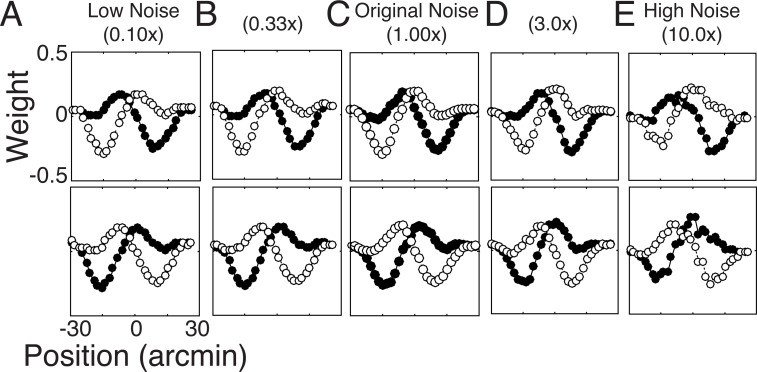
Effect of noise power on optimal filters. Optimal filters for 1/10^th^ the original noise variance, 1/3^rd^ the original noise variance, the original noise variance, 3x the original noise variance, and 10x the original noise variance. The filters are largely robust to substantial changes in noise variance.

Fig 11 shows that the optimal filters are largely robust to substantial changes in response noise variance. Specifically, the correlations between the filters in C (original noise variance) and the filters in A-E are 0.99, 0.99, 1.00, 0.98, and 0.90, respectively. The filters are nearly unchanged for a 30-fold change in noise (Fig 11A–11D). Increasing noise variance by a factor of 10, however, starts to break things down (*α* = 13.6 and $\sigma_{0}^{2}=2.30$; Fig 11E). This result should cut not come as a surprise. From classic ideal observer theory on target detection and discrimination [46–48], a proportional increase in noise will lower overall performance, but in general it will not change the optimal receptive field shapes. Thus, if the filters are learned with noise parameters that are ‘in the ballpark’ of the noise characteristics of neurons in cortex, the estimated filters should be near optimal for neurons in cortex even if the estimated noise parameters are off by some amount.

#### The effect of the cost function

Here, we examine the effect of changing the cost function that is used to learn the optimal receptive fields. To isolate the effect of the cost function, the training set and all other model parameters were identical to those used for the main results in the paper. The only change was to use an L2 norm (squared error) cost function.

**Fig 12:**
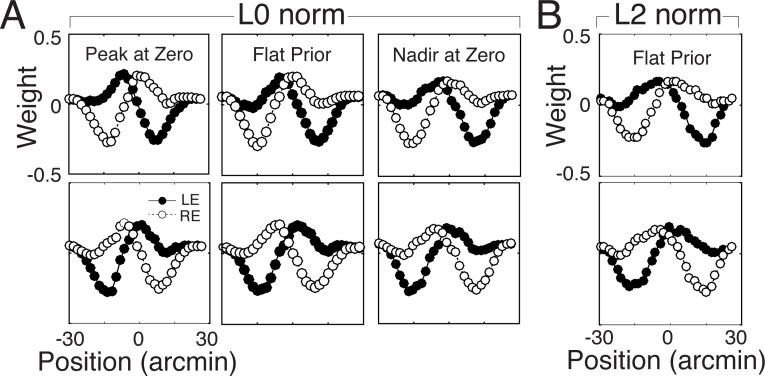
Effect of cost function on optimal filter shapes. **A** Optimal filters learned with L0 norm (KL divergence) cost function three different priors (c.f. Fig 10C, 10F and 10I). These priors correspond to the most extreme prior with a peak at zero, the flat prior, and the most extreme prior with a nadir at zero. **B** Optimal filters learned with the L2 norm (squared error) cost function and a flat prior. The L2 norm cost function has a subtle but systematic effect on the optimal filters. that is similar to the effect of a prior with excess mass at non-zero values.

Changing the cost function has a minimal effect on the optimal encoding filters in this task (Fig 12), just as changing the prior and noise power have minimal effects on the optimal encoding filters. The L2 norm cost function yields filters that are most similar (*ρ* = 0.95) to the L0 norm filters learned with a prior having excess mass at non-zero values. Again, this result should perhaps not be a surprise. Just as changing the cost function from L0 to L2 norm increases the penalty assigned to large errors, increasing the prior probability mass at non-zero values increases the importance of making fewer errors at those latent variable levels.

It is advantageous that the filters are generally robust to the different factors considered here (i.e. the prior, response noise power, and cost function). It suggests that for biologically plausible noise parameters, natural stimulus properties and the task of interest are the primary determinants of the filters that optimize performance in the task. This result is sensible: the properties of the stimulus should primarily determine the most useful receptive field shapes for extracting task relevant information from the stimuli.

## Discussion

Accuracy Maximization Analysis (AMA) is a method for task-specific dimensionality reduction that has contributed to the development of ideal observers for particular sensory-perceptual tasks in early- and mid-level vision [21–24]. It returns the encoding filters (receptive fields) that select the most useful information in proximal stimuli for estimating the value of a latent variable relevant for the task. In conjunction with psychophysical experimental techniques and carefully collected databases of natural images and scenes, the method has helped shed light on the fundamental computations that might be performed by the visual system in the service of particular tasks. Unfortunately, the method has a computational cost high enough as to render the method impractical for many purposes.

To improve the compute time, we derived the gradient for AMA and developed a batch stochastic gradient descent routine to increase the rate at which optimal task-specific filters can be learned. This method, AMA-SGD, finds the optimal filters in compute time that is linear, rather than quadratic, in the number of elements in the training set. In the process, we recognized that filters learned with batches with very few stimuli per level of the latent variable tend to be non-representative. AMA-SGD must therefore be used with caution. However, as our empirical demonstrations make clear, the benefits associated with AMA-SGD greatly outweigh its minor drawbacks, and make AMA a more practical tool for research in perception science.

In what follows, we contrast AMA and AMA-SGD with other methods for dimensionality reduction and neural characterization that provide encodings that are unique only up to a subspace spanned by a set of encoding filters. AMA has the potential to return not only the subspace, but the particular basis elements defining the subspace. This feature of the method is due to the interacting effects of filter correlation and response noise. Scaled additive (e.g. Poisson-like) response noise and non-orthogonal (correlated) receptive fields are widely documented features of neural systems. Many methods for dimensionality reduction and neural characterization are constrained to consider orthogonal filters only [1,49–52] [53], and/or have response models that assume encodings that are noiseless or are corrupted by constant additive noise only [49,50,54–58].

We find that scaled additive response noise tends to provide an encoding advantage over orthogonal filters with constant additive noise. We conclude by proposing a novel use for AMA. Specifically, we speculate that, if repurposed for the task of obtaining a descriptive model of the feature space driving a neuron’s response, AMA may be able to overcome a fundamental limitation of standard subunit models for neural characterization that prevents links from being established between model components and their biophysical analogs.

### Encoding Fidelity and Uniqueness within a Subspace

Standard forms of the most popular methods for dimensionality reduction (e.g. PCA) and statistical characterization (e.g. ICA) do not include a specific model of encoding noise. In such models, any set of receptive fields (i.e. basis elements) spanning the same subspace encode an arbitrary stimulus with equivalent fidelity. In other words, the encoding provided by a given pair of filters within their spanned subspace is not unique. This fact is due to an assumption common to a large class of popular methods for dimensionality reduction: namely, that the filters encode stimuli noiselessly.

**Fig 13:**
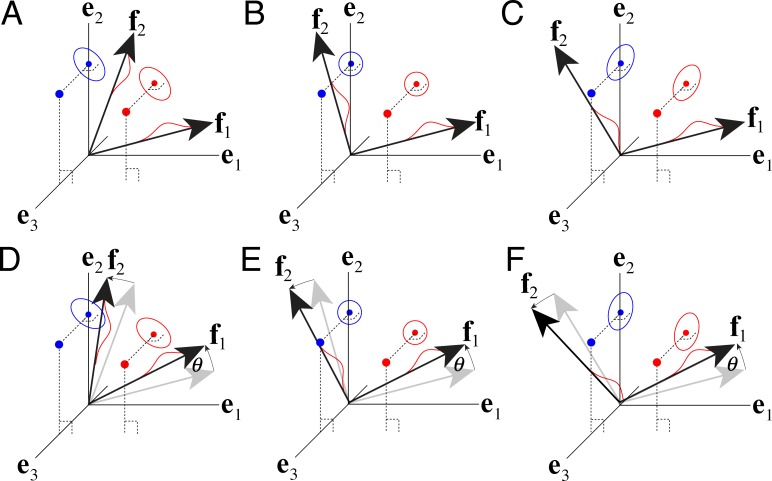
Stimulus encoding fidelity and uniqueness with constant additive noise. The original stimuli are represented as points in a three dimensional space (bigger red and blue dots, one stimulus from each of two levels of the latent variable). The original stimuli are then projected into a standard (i.e. orthogonal) basis {**e**_1_,**e**_2_} that spans the same subspace as two (possibly non-orthogonal) filters {**f**_1_,**f**_2_}. This subspace lies in the **e**_1_,**e**_2_ plane. The ellipse represents uncertainty about each encoded stimulus. The size and orientation of each uncertainty ellipse is determined by the stimulus (red dot), each filter’s response noise, and correlation between the filters. Red Gaussian bumps represent the noisy response distributions of F1 and F2 to the red stimulus. **A** Positively correlated ($\rho =f_{1}^{T}f_{2}>0$) filters. **B** Orthogonal (i.e. uncorrelated; $\rho =f_{1}^{T}f_{2}=0$) filters. **C** Negatively correlated ($\rho =f_{1}^{T}f_{2}<0$) filters. **D-F** Rotated versions of A-C. Orthogonal filters (B,E) provide rotation invariant encoding; non-orthogonal (i.e. positively and negatively correlated) filters do not (A,C,D,F).

**Fig 14:**
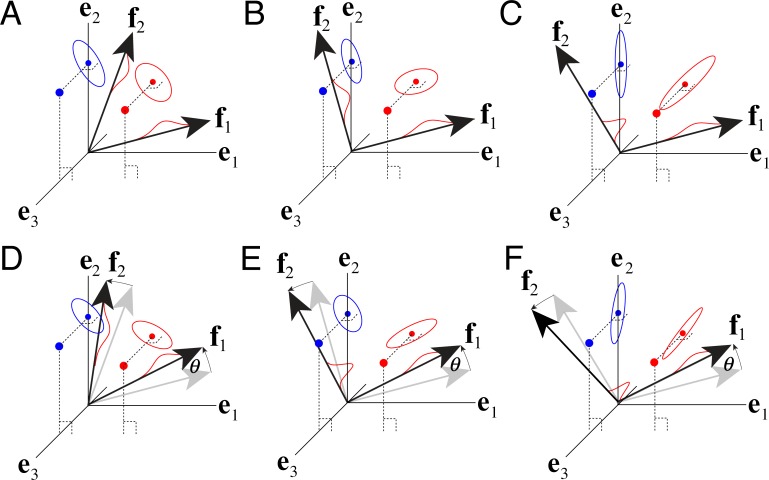
Stimulus encoding fidelity and uniqueness with scaled additive noise (i.e. additive noise with response variance multiplicatively related to the response mean). The original stimuli are represented as points in a three dimensional space (bigger red and blue dots, one stimulus from each of two levels of the latent variable). The original stimuli are then projected into a standard (i.e. orthogonal) basis {**e**_1_,**e**_2_} that spans the same subspace as two (possibly non-orthogonal) filters {**f**_1_,**f**_2_}. The uncertainty ellipse represents uncertainty about the encoded stimulus given the filter responses. The size and orientation of each uncertainty ellipse is determined by the stimulus (dot), the filter response noise, and the correlation between the filters. **A-F** Unlike with constant additive noise, stimulus encoding with scaled additive noise is unique (up to a sign flip) regardless of whether the filters are orthogonal. Filters that are somewhat anti-correlated yield uncertainty ellipses that are oriented approximately with lines radiating from the origin.

Encoding noise corrupts measurements by real biological or machine vision systems. AMA incorporates an explicit noise model at the level of the encoding filters (Eqs 1a–1d), as do probabilistic extensions to PCA and ICA[50,58]. Encoding noise (i.e. the filter response model) can make the stimulus encoding unique within the subspace that the encoding filters define. Figs S1, 13 and 14 are designed to help develop a geometric intuition for why filter response noise can make the encoding of particular filters within a subspace unique. After building intuition, we discuss the implications of this fact for our understanding of neural coding. We consider three classes of encoding filter response: i) a noiseless response model (*α* = 0 and $\sigma_{0}^{2}=0$, see Eq 1d; S1 Fig) ii) an constant additive response model (*α* = 0 and $\sigma_{0}^{2}>0$; Fig 13), and iii) a scaled additive response noise model (*α* > 0 and $\sigma_{0}^{2}>0$); Fig 14). We also consider the impact of having orthogonal (i.e. uncorrelated) encoding filters vs. non-orthogonal (i.e. correlated or anti-correlated) encoding filters. We will see that the type of noise (constant additive or scaled additive), filter correlation (i.e. redundancy), and filter orientation in the subspace can interact non-trivially to confer coding advantages.

Consider two stimuli that are projected into a standard basis spanned by an arbitrary pair of filters **f**; let this subspace be represented by orthonormal basis **e** (Figs S1, 13 and 14) If noiseless encoding is assumed (which is of course biologically unrealistic), the stimuli are encoded with equal fidelity no matter the filter correlation (redundancy) or rotation, so long as the filters lie in the same subspace. Specifically, filters F1 and F2 encode the stimulus identically well, regardless of whether the encoding filters are positively correlated, orthogonal, or anti-correlated. Rotating the encoding filters in the subspace also has no impact on coding fidelity. Thus, with no encoding noise, every set of filters spanning the same subspace provides an equivalent stimulus encoding (S1 Fig).

With constant additive response noise the situation changes. Now, filters F1 and F2 encode the stimulus with different fidelity when they are correlated vs. when they are orthogonal; note the differences in the uncertainty ellipses (Fig 13A–13C). When the filters are orthogonal (Fig 13B and 13E), the uncertainty ellipses are circular, and stimulus encoding remains invariant to rotation (Fig 13E). Stimulus encoding by correlated filters, however, is no longer invariant to filter rotation (Fig 13A, 13D, 13C and 13F).

With scaled additive response noise, the situation changes still further. Filters F1 and F2 now provide a unique encoding of the stimulus, regardless of whether they are correlated or uncorrelated, and regardless of whether the filters are rotated or not (Fig 14A–14F).

The fact that the fidelity of stimulus encoding changes as a function of filter correlation and rotation within a subspace suggests that encoding cost (i.e. the value of AMA objective function) may depend on the particular filters within a given subspace. To examine this issue quantitatively, we rotated the optimal receptive field pair within their spanned subspace and computed the cost for each rotation angle *θ*. (see S8 Text). Recall that the optimal filters in the current task are somewhat anti-correlated ($\rho =f_{1}^{T}f_{2}=-0.22$). We also examined the cost of forcing the filters to be orthogonal. To do so, we performed Gram Schmidt orthogonalization, rotated the orthogonalized filters, and computed the cost for each rotation angle *θ*.

**Fig 15:**
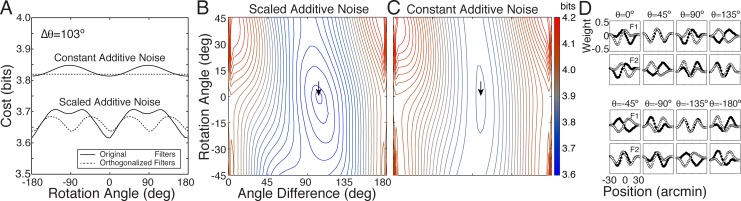
Encoding cost in the subspace spanned by the filters. **A** Cost as a function of rotation angle for response noise models with scaled additive and constant additive noise. With scaled additive noise, the optimal filters (lower solid curve) provide a unique encoding up to a sign flip (i.e. rotation angle = 180°). Orthogonal filters with scaled additive noise that span the same subspace (lower dashed curve) provide an encoding that is periodic on 90°. For comparison, cost as a function of rotation angle for filters with constant additive noise and matched noise power is also shown (see text). (Note that the original, optimal filters (c.f. Fig 6A) have a cosine similarity (i.e. correlation) of *ρ* = -0.22, corresponding to an angle difference of 103°.) **B** Cost landscape for scaled additive noise within the subspace spanned by filters 1 and 2 for all possible rotation angles and angle differences (i.e. correlations). The curves in A show vertical slices through this space. Arrow marks optimal filters. **C** Cost landscape with additive noise. **D** Filters as a function of rotation angle in the subspace.

Fig 15 shows that some filter pairs within the subspace yield lower cost than others (Fig 15A–15C). Example filter pairs that have been rotated by different amounts are depicted in Fig 15D. With scaled additive response noise (the noise model with which the filters were learned), cost is lowest for the optimal filters. For all non-zero rotation angles cost increases, except for 180° (Fig 15A). (A 180° rotation angle corresponds to contrast reversal of both receptive fields, which by assumption (Eqs 1a–1d), gives identical performance to the original filters). If the filters are orthogonalized, cost increases on average. More importantly, the minimum cost of the best pair of orthgonalized filters is higher than the minimum cost of the original somewhat anti-correlated filters. This result shows that correlated filters can provide a coding advantage over orthogonal filters in the AMA framework.

Next, we examined constant additive response noise models. With constant additive (instead of scaled additive) response noise, cost is also modulated by the rotation angle within the subspace, but only if the filters are non-orthogonal. If the filters are orthogonal, all filter rotations within the subspace provide an identical encoding (Fig 15A). These results are consistent with the intuitions developed in Fig 13. To make a quantitative comparison between the encoding costs associated with the two noise models, we matched the noise power between the two models. Specifically, we set the constant additive noise variance equal to the average variance of the scaled additive noise $\sigma_{constant}^{2}=\frac{1}{N}\sum_{kl}\sigma_{scaled}^{2}$ where *N* is the total number of stimuli in the training set. Encoding filters having this constant additive noise never achieve costs as low as the scaled additve noise model (Fig 15A and 15C). So long as noise power is matched, this result holds whether the filters are learned with scaled additive or constant additive noise. Therefore, for the task considered here (disparity estimation), scaled additive noise provides a coding advantage.

This same result holds for several other fundamental tasks in early vision with natural images (retinal speed estimation, motion-in-depth estimation). These tasks have all been successfully modeled with energy-like computations (disparity energy model, motion energy model, etc.). We conclude that scaled additive noise provides a coding advantage over constant additive noise in an important class of estimation tasks in early- and mid-level vision for which energy-like computations are appropriate.

There are several take-away points. First, in AMA, all encoding filters, even those spanning the same subspace, do not provide equivalent encodings. Second, correlated filters can yield lower cost encodings than orthogonal filters. Third, scaled additive response noise can yield lower cost encodings than constant additive response noise when the noise power (i.e. average noise variance) is matched. These results have implications for how to think about the pros and cons of the constraints imposed on many methods for dimensionality reduction.

### Scaled Additive Neural Noise and Filter Correlation

In this section, we examine why scaled additive response noise can provide an advantage over constant additive response noise. In early visual areas, neural response variance increases approximately linearly with the mean response [31,32]. Much attention has been paid to this property of neural response, especially as it relates psychophysical performance in target detection, a paradigmatic task in the spatial vision literature. When response variance is proportional to the mean response, a single neuron’s signal-to-noise ratio for detection of a particular target is proportional to the square-root of the mean response, $SNR\propto \sqrt{r}$. On the other hand, if neural response variance is independent of the mean response (i.e. constant), the signal to noise ratio is proportional to the mean response, *SNR* ∝ *r*. Thus, it has been sensibly argued that, all other things equal, scaled additive noise must have deleterious effects on neural coding compared to constant additive noise.

**Fig 16:**
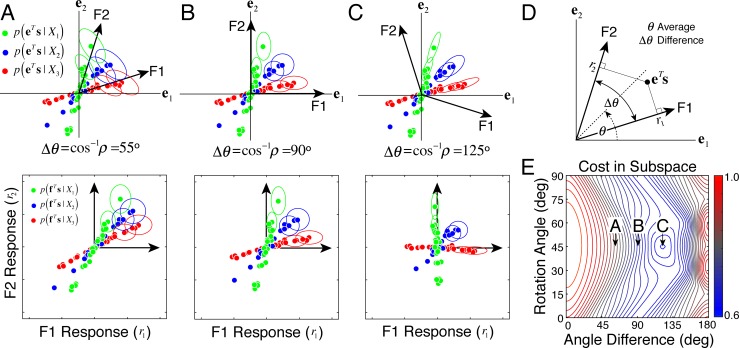
Filter correlation, scaled additive noise, and effects on stimulus encoding. **A-C** Conditional stimulus distributions, projected into the subspace spanned by the filters, represented two ways. Upper row: stimulus distributions *p*(**e**^*T*^**s**|*X*_*i*_) conditioned on different values of the latent variable (red, green, blue) projected into the subspace spanned by the filters. The cardinal axes in the standard basis (*e*_1_ and *e*_2_) are orthonormal by definition whereas the filters are not necessarily orthogonal. Lower row: conditional filter response distributions. Changing the correlation between the filters from positive (A), to orthogonal (B), to anti-correlated (C) alters how the uncertainty ellipses are aligned with the stimulus distributions in the standard basis. **D** Definition of rotation angle and angle difference. **E** Cost landscape in the subspace defined by the filters. The minimum occurs for the situation in C when the filters are anti-correlated (angle difference > 90°). The interaction with scaled additivenoise causes the uncertainty ellipses to be maximally aligned with the stimulus distributions.

However, in the case considered above, scaled additive noise (response noise variance proportional to mean response) outperforms constant additive noise with matched noise powers. Many (most?) visual tasks are performed at super threshold contrasts and involve estimating the value of a variable that is latent in the proximal stimulus. In latent variable estimation and discrimination tasks, scaled additive noise can benefit, rather than deteriorate the quality of neural encoding. This is true in the current task of disparity estimation (Figs 6 and 14–16). It is also true of other related tasks in early vision (e.g. speed estimation and motion-in-depth estimation). This result raises the possibility that a ubiquitous neural response property that hurts performance in contrast detection tasks may actually benefit performance in tasks that are somewhat ‘higher-level’ (e.g. disparity estimation).

Why, in latent variable estimation and discrimination, can a vision system with scaled additive noise outperform a vision system with matched constant additive noise? Some development is necessary to answer this question because the answer depends on a set of interlocking dependencies between the noise model, filter correlations, and the latent-variable-conditioned stimulus distributions *p*(**s**|*X*_*i*_). When the task is to discriminate one latent variable value from another (as opposed to detecting a well-defined contrast pattern—a signal-known-exactly task), it is less clear what constitutes ‘signal’ and what constitutes ‘noise’. We have found it useful to approach the problem with standard techniques in the pattern classification literature [59].

Consider a hypothetical case that illustrates the relevant principles. Fig 16A–16C shows three simulated stimulus distributions projected into the subspace spanned by a pair of filters. (These simulated distributions are superficially similar to the disparity conditioned stimulus distributions shown in Fig 6C.) These same exact stimuli are encoded by three pairs of filters that are differently correlated, but that have the same scaled additive noise and that span the same subspace (Eqs 1a–1c). This subspace is represented by the orthonormal basis **e** that spans the same subspace as the filters **f**. The upper and lower rows of Fig 16A–16C represent the same information in different forms. In the upper row, three latent-variable-conditioned stimulus distributions are projected into the subspace defined by a pair of filters *p*(**e**^*T*^**s**|*X*_*i*_); the dots represent the stimulus projections in the standard basis and the ellipses represent encoding uncertainty. In the lower row, the exact same stimulus projections are represented by the mean responses that they elicit from each filter pair, *p*(**f**^*T*^**s**|*X*_*i*_); the dots represent the stimulus projections onto the filters and the ellipses represent filter response noise. We refer to the lower row as the *filter basis*. Consistent with the assumption that the filter response noise is independent (Eqs 1c and 1d), all the noise ellipses in the filter basis (lower row) are aligned with the axes of the space (i.e. the noise covariance matrix is diagonal). The linear mappings from the filter basis to the standard basis and back are derived in S8 Text. The oblique orientations of the uncertainty ellipses in the upper row of 16ac therefore *do not* reflect noise correlations [60,61].

Now, examine the effect of changing the filters from being positively correlated (Fig 16A), to orthogonal (Fig 16B), to anti-correlated (Fig 16C). As filter correlation and orientation within the subspace changes (see Fig 16D), the uncertainty ellipses (upper row) change their orientation. Cost is minimized when the uncertainty ellipses maximally align with the projections of the conditional stimulus distributions (Fig 16E). Remarkably, filter correlation (i.e. cosine similarity), filter orientation in the subspace, and scaled additive noise can conspire to align the uncertainty ellipses with the conditional stimulus distributions.

The conditional distributions of filter responses are shown in the lower row of Fig 16A–16C. The mean filter response to each stimulus is obtained by projecting the stimulus onto each filter (c.f. Fig 16D; **r** = **f**^*T*^**s**). In filter response space (i.e. the filter basis), two effects occur as filter correlation changes. The most dramatic effect is the change in the distribution the response means. A secondary effect is that the height and/or width of the response noise ellipses decrease as the corresponding mean filter response approaches zero. Note, however, that the noise ellipses always remain aligned with the cardinal axes. In other words, the noise ellipses have diagonal covariance matrices, consistent with the assumption of independent response noise. In filter response space, the algorithm’s aim is to position the filters such that the conditional response distributions *p*(**R**|*X*_*i*_) are as discriminable from each other as possible.

With a constant additive noise model, in the standard basis, all uncertainty ellipses have the same orientation (c.f. Fig 13B); in the filter basis, all noise ellipses are circular (i.e. equal variance diagonal covariance matrices). Thus, if the conditional stimulus distributions change orientation as a function of the value in the latent variable (as they do here), the constant additive noise model cannot align the uncertainty ellipses with the stimulus distributions across the space. As a consequence, encoding cost increases (Fig 15A, S2 Fig).

In general, cost is minimized when encoding uncertainty is maximized within, and minimized between, latent-variable-conditioned stimulus distributions. That is, when uncertainty due to noise maximally overlaps the uncertainty due to ‘nuisance’ stimulus variation, coding of the latent variable is improved. A related claim about the potential utility of noise correlations has recently been made [61,62]. Additionally, scaled additive noise yields lower response variance for stimuli near the origin than stimuli far from the origin of the response space, thereby reducing the relative cost of ‘hard’ stimuli and increasing the relative cost of ‘easy’ stimuli. Each stimulus therefore contributes more evenly to the cost. In the tasks considered, this property causes the algorithm to make better use of the information provided by each stimulus. If the expressions underlying AMA were reformulated as a learning rule, we suspect that scaled additive noise would enable the system to learn more efficiently. Most importantly, we have shown that scaled additive noise and non-orthogonal filters can confer significant benefits to neural encoding. These benefits are obtained when uncertainty due to noise is shaped to match within-level stimulus variation.

### Limitations and Future Directions

The AMA cost landscape is non-convex, so there is no guarantee that the filters found by the algorithm indeed represent the global minimum. However, there are several reasons to suspect that the filters for disparity estimation presented here indeed found a global minimum. First, the recovered filters occupy the minimum of the cost landscape within the subspace that they span (Fig 15A–15C). Second, somewhat surprisingly, correlated (non-orthogonal) filters with scaled additive noise tend yield lower cost landscapes with deeper minima (Fig 15B and 15C) than orthogonal filters with constant additive noise. Third, the work presented here and in previous publications has found that different random initializations tend to yield equivalent filters.

The response model used here allows both positive and negative encoding filter responses whereas real neurons give only positive responses. Future work will examine the pros and cons of incorporating half-rectification into the response model (Eqs 1a–1d). One drawback of incorporating half-rectification is that more filters will be required to cover the same response space, thereby increasing the dimensionality of the search space, perhaps leading to less stable performance. However, incorporating half-rectification will increase biological realism, allow for differential sensitivity to ON/OFF contrast changes [63], and increase the flexibility of the system to match stimulus encoding uncertainty to task-irrelevant stimulus variation (Figs 13–16).

### AMA for Neural Systems Identification

Interest in neural systems identification has surged in recent years. The field has generated a slew of models with ever increasing sophistication and descriptive power. Many of these models are known as ‘subunit models’. Subunit models seek to provide a computational level description of a neuron’s processing that can predict a neuron’s response to arbitrary stimuli.

The spike-triggered average (STA) and spike-triggered covariance (STC) analysis are early examples of subunit models [64–67]. The generalized linear model (GLM) and generalized quadratic model (GQM) are examples of more recently developed subunit models that are more flexible and powerful [68–70]. (All of these methods have been adapted to handle non-spiking, real-valued data (e.g. response rates or intracellular voltages[69,71]). These methods have been widely adopted by the neuroscience community because of their success in providing compact, interpretable characterizations of the input-output relationship between stimuli and neural response. In general, subunit models describe neural response with a low-dimensional set of stimulus features (i.e. subunit receptive fields), a nonlinear pooling rule, a static output non-linearity, and noise function that generates output noise. As these models have increased in descriptive power and mathematical elegance, interest has increased in whether the computational components can be mapped back to specific biophysical components. For example, in a subunit model description of a complex cell, one may ask whether presynaptic simple cells are the biophysical analogs of the model subunits.

A limitation of this class of subunit models is that although they can recover the subspace spanned by a set of receptive fields, the models cannot recover the subunit receptive fields themselves. In traditional subunit models, any set of receptive fields spanning the same subspace encodes a given stimulus with equal fidelity. This property of subunit models is due to the fact that they implicitly assume noiseless encoding. AMA, on the other hand, has an explicit model of response noise for each filter (i.e. subunit receptive field). As discussed above, response yields encodings that are unique within the subspace defined by the filters (Figs 15, 16 and S1). By adapting AMA as a method for neural system’s identification, we speculate that it may be possible to identify both the subspace spanned by the subunit receptive fields, and the individual subunit receptive fields themselves. As neural datasets come online having simultaneous recordings between ‘target cells’ and their presynaptic inputs (e.g. connected V1 and LGN units), these possibilities can be tested explicitly.

Explicitly modeling noise at the level of the subunit receptive field responses does not come without its own set of drawbacks. The GLM and GQM have cost landscapes that are convex; the local minimum is guaranteed to be the global minimum under the model. The cost landscape in AMA is non-convex, so guarantees cannot be made that the minima found via AMA are global minima. However, in the cases we have examined (see above), AMA results tend to be stable. Future work must determine whether this research direction is viable, but the ingredients are there to justify searching for a productive way forward.

### Conclusions

This manuscript presents technical improvements to and conceptual insights about Accuracy Maximization Analysis (AMA), a recently developed Bayesian method for task-specific dimensionality reduction [22]. The manuscript has four primary aims. First, it provides a thorough and intuitive review of AMA, explaining the logic behind method’s setup and its solutions. Second, it contributes two technical advances- the gradient of the cost function and a stochastic gradient descent routine- that markedly decrease compute time, thereby making it a more practical tool for research in sensation and perception. Third, it shows that the effects of the prior over the latent variable, internal noise, and the cost function can be examined relative to the effect of stimulus variability. Fourth, it examines several non-standard features of the method—its ability to model scaled additive noise and learn correlated filters—that make it more flexible than other more widely known methods. This flexibility confers a coding advantage, and renders the method capable of identifying particular filters (receptive fields) within the subspace that they span. This capability is due primarily to the explicit modeling of noise at the level of the encoding filter responses, which all biological systems suffer from. Perceptual psychology and visual neuroscience are relatively young fields, but they are advancing rapidly, and cross-pollination between the sub-disciplines is increasingly common. As research with natural stimuli becomes increasingly common, widespread application of this method may help speed progress.

## Supporting Information

S1 Fig Stimulus encoding and uniqueness without filter response noise. (PDF)

S2 Fig Filter correlation, constant additive noise, and effects on stimulus encoding. (PDF)

S3 Fig Comparison of ICA, PCA, and AMA filters in a simulated case. (PDF)

S1 Text Posterior probability distribution over the latent variable. (PDF)

S2 Text AMA gradient with the 0,1/KL-divergence cost function. (PDF)

S3 Text AMA gradient with the squared error cost function. (PDF)

S4 Text Optimal estimator for 0,1 cost function is MAP estimator. (PDF)

S5 Text KL-divergence is negative log-probability of correct latent variable. (PDF)

S6 Text Posterior mean is optimal estimator for squared error cost function. (PDF)

S7 Text Rotating correlated filters within the spanned subspace. (PDF)

S8 Text Uncertainty ellipses for encoding with correlated filters in standard basis. (PDF)
