# Correction: Accuracy Maximization Analysis for Sensory-Perceptual Tasks: Computational Improvements, Filter Robustness, and Coding Advantages for Scaled Additive Noise

**DOI:** 10.1371/journal.pcbi.1005636

**Published:** 2017-07-10

**Authors:** The PLOS Computational Biology Staff

The published version of the manuscript contains a number of errors.

Please download a corrected version of the manuscript here: http://burgelab.psych.upenn.edu/research/Publications_files/BurgeJaini_PLoSCB_2017_Corrected.pdf

- There is an error on page 2. In the Author Summary, the last word of the first sentence should be ‘precedent’ instead of ‘precedence’. The sentence should read “In psychophysics and neurophysiology, the stimulus features that are manipulated in experiments are often selected based on intuition, trial-and-error, and historical precedent.”
- There are errors in Equations 2–5 on pages 6–7. The summation on the variable *i* in the denominator should have the range “*i* = 1 to *N*_*lvl*_” instead of the range “*i =* 1 to *N*_|*v*|_”.
- There is an error on page 14. In the Results section, the last sentence of the first paragraph should read “Finally, in the discussion section, we examine the general implications of these results for ……” instead of “Finally, in the discussion section, we examine the general implications of these results for …”.
- There is an error on page 19. In the section titled ‘The effect of noise power’, the last sentence of the first paragraph should begin “To isolate the effect of noise variance…” instead of “To isolate the effect of cut noise variance…”.
- There is an error on page 19. In the section titled ‘The effect of noise power’, the sentence beginning on the 5^th^ line of the second paragraph should read “This result should not come as a surprise.” instead of “This result should cut not come as a surprise.”.
