# Supplementary material for: Accuracy Maximization Analysis for Sensory-Perceptual Tasks: Computational Improvements, Filter Robustness, and Coding Advantages for Scaled Additive Noise
Source: PLoS Comput Biol. 2017 Feb 8;13(2):e1005281. doi: 10.1371/journal.pcbi.1005281 (PMC5298250; doi:10.1371/journal.pcbi.1005281)
Supplement: S1 Fig — (PDF) [file pcbi.1005281.s001.pdf]

# Supporting Information

## Accuracy Maximization Analysis for Sensory-Perceptual Tasks: Computational Improvements, Filter Robustness, and Coding Advantages for Scaled Additive Noise

Johannes Burge[1,2] & Priyank Jaini[1,3]

[1] Department of Psychology, University of Pennsylvania, USA
[2] Neuroscience Graduate Group, University of Pennsylvania, USA
[3] David R. Cheriton School of Computer Science, University of Waterloo, Canada

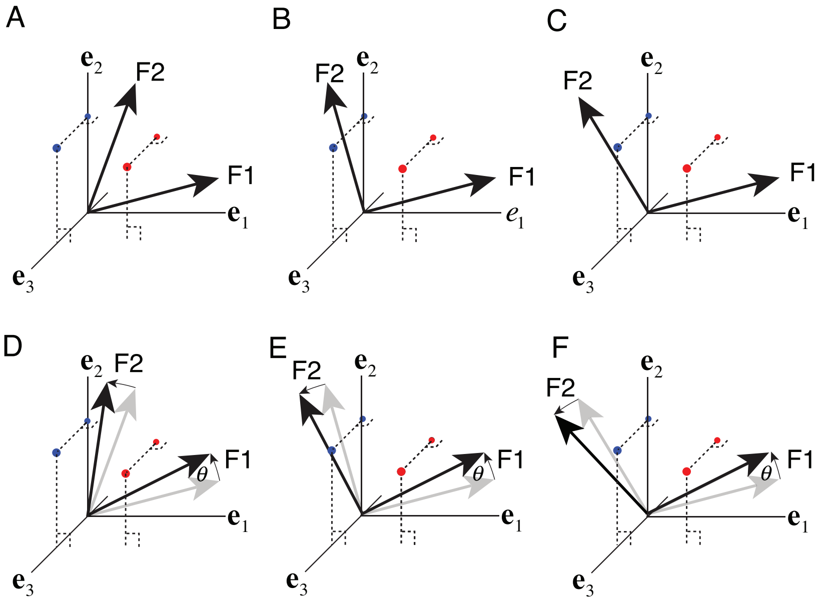


**Figure S1.** Stimulus encoding and uniqueness without filter response noise. The stimuli are represented as points in a three dimensional space (one stimulus from each of two levels of the latent variable, red and blue). The stimuli are projected into a standard basis spanned by two filters. Red Gaussian bumps represent individual filter encoding uncertainty for filter F1 and F2 to the red stimulus. When encoding is noiseless, encoding fidelity is invariant under changes in filter correlation (**A-C**) or filter rotation **(E-F)**. Thus, F1 and F2 provide identical encoding regardless of whether they are orthogonal or not, and whether they are rotated are not.
