# Supplementary material for: Accuracy Maximization Analysis for Sensory-Perceptual Tasks: Computational Improvements, Filter Robustness, and Coding Advantages for Scaled Additive Noise
Source: PLoS Comput Biol. 2017 Feb 8;13(2):e1005281. doi: 10.1371/journal.pcbi.1005281 (PMC5298250; doi:10.1371/journal.pcbi.1005281)
Supplement: S2 Fig — (PDF) [file pcbi.1005281.s002.pdf]

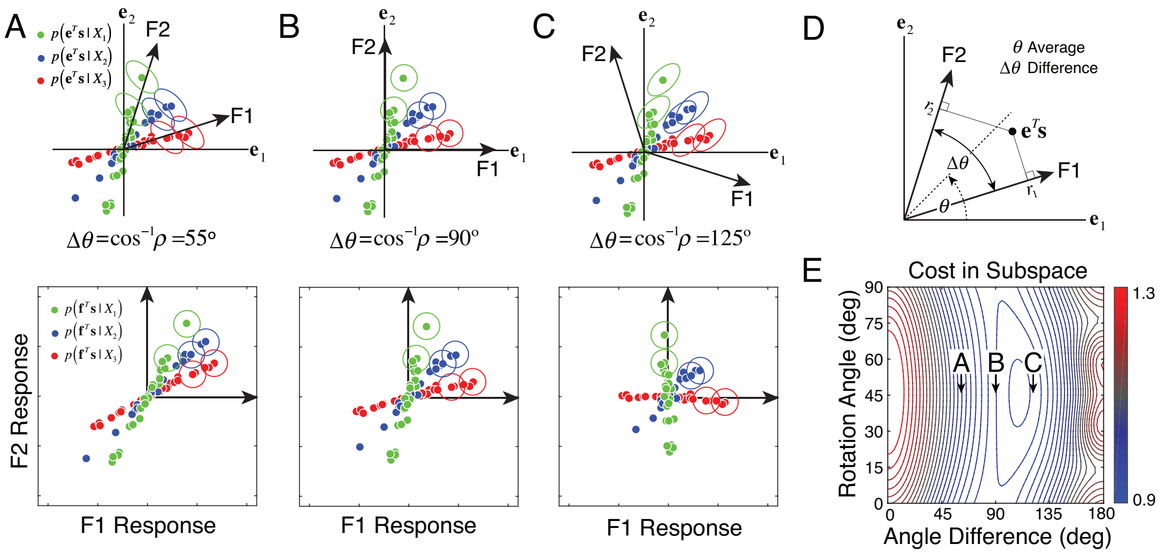

Figure S2. Filter correlation, constant additive noise, and effects on stimulus encoding. Conditions are identical as Figure 16 in the main text, except that the noise variance is constant with matched noise power. **A-C** Conditional stimulus distributions $p(\mathbf{e}^T\mathbf{s} \mid X_i)$ projected into the subspace spanned by the filters. Upper row: stimuli in a standard basis conditioned on different values of the latent variable. Lower row: filter response distributions. Changing the correlation between the filters from positive (A**)**, to orthogonal (B**)**, to anti-correlated (C) alters how the uncertainty ellipses are aligned with the stimulus distributions. **D** Definition of rotation angle and angle difference. **E** Cost landscape in the subspace defined by the filters. The minimum occurs when the filters are anti-correlated (angle difference > 90º). Constant additive noise forces all uncertainty ellipses to have the same orientation in the standard basis. Thus, they cannot align well with the conditional stimulus distributions. Overall cost is higher than with scaled additive noise having matched noise power (c.f. Fig. 15).
