# Supplementary material for: Accuracy Maximization Analysis for Sensory-Perceptual Tasks: Computational Improvements, Filter Robustness, and Coding Advantages for Scaled Additive Noise
Source: PLoS Comput Biol. 2017 Feb 8;13(2):e1005281. doi: 10.1371/journal.pcbi.1005281 (PMC5298250; doi:10.1371/journal.pcbi.1005281)
Supplement: S3 Fig — (PDF) [file pcbi.1005281.s003.pdf]

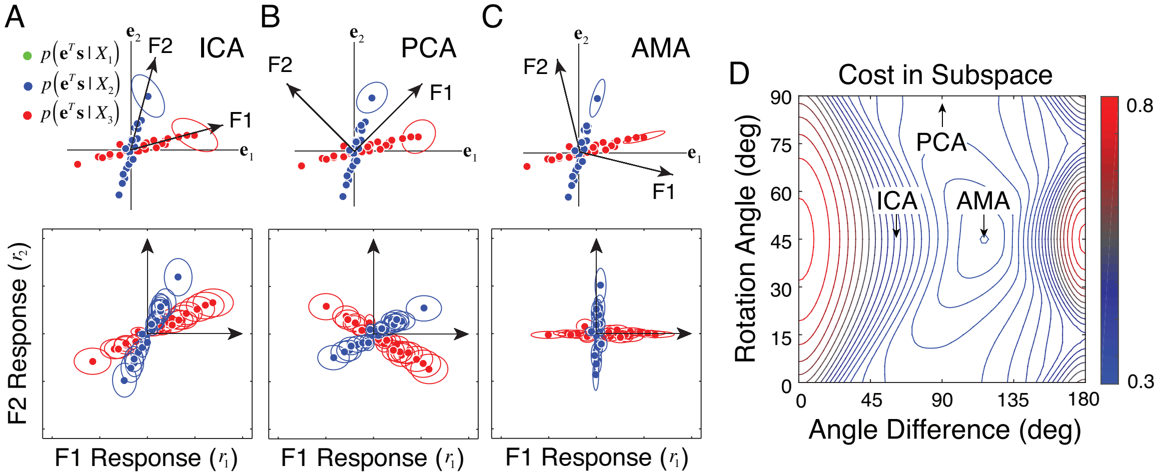

Figure S3. Comparison of ICA, PCA, and AMA filters in a simulated case with two latent variable levels and multiple stimuli per level. The different aims of the different routines cause different filters to be selected. **A** ICA filters, **B** PCA filters, and **C** AMA filter for the simulated dataset. Upper and lower rows show the stimuli in the standard basis and in the filter basis. Note the difference between the AMA filters and those returned by the other routines. ICA finds the directions along which the marginal stimulus projections have the highest kurtosis. PCA find the directions for which the stimulus projections have maximum variance. AMA finds the directions that allow the latent variable to be decoded as accurately as possible. **D** Cost in subspace defined by these receptive fields. It is no surprise that AMA outperforms the ICA and PCA filters in this simulated example.
