# Supplementary material for: Accuracy Maximization Analysis for Sensory-Perceptual Tasks: Computational Improvements, Filter Robustness, and Coding Advantages for Scaled Additive Noise
Source: PLoS Comput Biol. 2017 Feb 8;13(2):e1005281. doi: 10.1371/journal.pcbi.1005281 (PMC5298250; doi:10.1371/journal.pcbi.1005281)
Supplement: S1 Text — (PDF) [file pcbi.1005281.s004.pdf]

S1 Text: Posterior probability distribution over the latent variable

Here, following Geisler et al (2009), we derive the expression for the posterior probability of X_k given a labeled training set of stimuli. The levels (or values) of the latent variable are indexed by k and i. The stimuli having levels k and i are indexed by m and j, respectively. The index l denotes a particular stimulus within level k, and is the stimulus for which the posterior probability distribution over the categories is being computed. The particular filter within the filter set is indexed by t. The posterior probability for a particular value of the latent variable X_k was derived in the main text (equation 5)

$$P\left(X_k \mid \mathbf{R}(k,l)\right)=\frac{\sum_{m=1}^{N_k} P\left(\mathbf{R}(k,l) \mid \mathbf{s}_{km}\right)}{\sum_{i=1}^{N_{lvl}}\sum_{j=1}^{N_i} P\left(\mathbf{R}(k,l) \mid \mathbf{s}_{ij}\right)}$$

Assuming that the filter responses are conditionally independent (which identical to assuming that the noise correlations are zero)

$$P\left(X_k \mid \mathbf{R}(k,l)\right)=\frac{\sum_{m=1}^{N_k}\prod_{t=1}^{N_q} P\left(R_t(k,l) \mid \mathbf{s}_{km}\right)}{\sum_{i=1}^{N_{lvl}}\sum_{j=1}^{N_i}\prod_{t=1}^{N_q} P\left(R_t(k,l) \mid \mathbf{s}_{ij}\right)}$$

Assuming the filter response is corrupted by scaled additive Gaussian noise (i.e. additive Gaussian noise with variance proportional to the mean; equations 1a-d in the main text), the posterior probability X_k given a noisy response to a stimulus $\mathbf{s}_{kl}$ is

$$P\left(X_k \mid \mathbf{R}(k,l)\right)=\frac{\sum_{m=1}^{N_k}\left(\prod_{t=1}^{N_q}\sigma_{km,t}\right)^{-1}\exp\left[-\frac{1}{2}\sum_{t=1}^{N_q}\left(\frac{R_t(k,l)-r_{km,t}}{\sigma_{km,t}}\right)^2\right]}{\sum_{i=1}^{N_{lvl}}\sum_{j=1}^{N_i}\left(\prod_{t=1}^{N_q}\sigma_{ij,t}\right)^{-1}\exp\left[-\frac{1}{2}\sum_{t=1}^{N_q}\left(\frac{R_t(k,l)-r_{ij,t}}{\sigma_{ij,t}}\right)^2\right]} \tag{S1}$$

The posterior probability of X_k given the expected (i.e. mean) response to stimulus $\mathbf{s}_{kl}$ is

$$P\left(X_k \mid \mathbf{R}(k,l)\right)=\frac{\sum_{m=1}^{N_k}\left(\prod_{t=1}^{N_q}\sigma_{km,t}\right)^{-1}\exp\left[\overbrace{-\frac{1}{2}\sum_{t=1}^{N_q}\left(\frac{r_t(k,l)-r_{km,t}}{\sigma_{km,t}}\right)^2}^{\Delta_{km}(k,l)}\right]}{\sum_{i=1}^{N_{lvl}}\sum_{j=1}^{N_i}\left(\prod_{t=1}^{N_q}\sigma_{ij,t}\right)^{-1}\exp\left[\underbrace{-\frac{1}{2}\sum_{t=1}^{N_q}\left(\frac{r_t(k,l)-r_{ij,t}}{\sigma_{ij,t}}\right)^2}_{\Delta_{ij}(k,l)}\right]} \tag{S2}$$
