# Supplementary material for: Accuracy Maximization Analysis for Sensory-Perceptual Tasks: Computational Improvements, Filter Robustness, and Coding Advantages for Scaled Additive Noise
Source: PLoS Comput Biol. 2017 Feb 8;13(2):e1005281. doi: 10.1371/journal.pcbi.1005281 (PMC5298250; doi:10.1371/journal.pcbi.1005281)
Supplement: S2 Text — (PDF) [file pcbi.1005281.s005.pdf]

S2 Text: AMA gradient with the 0,1/KL-divergence cost function
The total cost for a set of filters is given by the average expected cost across all stimuli

$$\bar{C} = \frac{1}{N}\sum_{kl}^{N} E_{\mathbf{R}(k,l)}\left[C_{kl}\right]$$

Given the 0,1 cost function, the cost associated with the filter response to an arbitrary stimulus is given by 1.0 minus the posterior probability at the correct latent variable value $C_{kl} = 1 - P\left(X_k \mid \mathbf{R}(k,l)\right)$ (see S4 Text). This cost is closely related to the KL-divergence (see S5 Text) which we refer to, in a slight abuse of terminology, as the KL-divergence cost. Thus,

$$C_{kl} = -\log P\left(X_k \mid \mathbf{R}(k,l)\right)$$

Using the approach of Geisler et al (2009), we approximate the expected cost for each stimulus with the cost given the expected response. Thus,

$$\begin{aligned} E_{\mathbf{R}(k,l)}\left[C_{kl}\right] &= -\int_{-\infty}^{\infty} \log P\left(X_k \mid \mathbf{R}(k,l)\right) P\left(\mathbf{R}(k,l) \mid \mathbf{s}_{kl}\right) d\mathbf{R}(k,l) \\ &\cong -\log P\left(X_k \mid \mathbf{r}(k,l)\right) \end{aligned}$$

The gradient for the overall cost (under the approximation) is thus given by

$$\nabla_{\mathbf{f}_q}\bar{C} = -\frac{1}{N}\sum_{kl}^{N}\left[\nabla_{\mathbf{f}_q} \log p\left(X_k \mid \mathbf{r}(k,l)\right)\right]$$

To simplify the subsequent derivation, we perform variable substitutions in the expression for the posterior probability. First, we define $\Delta_{km}(k,l)$ and $\Delta_{ij}(k,l)$ as the exponentiated terms in the numerator and denominator of equation S2, to obtain

$$P\left(X_k \mid \mathbf{R}(k,l)\right) = \frac{\overbrace{\sum_{m=1}^{N_k} \overbrace{\left(\prod_{t=1}^{N_q} \sigma_{km,t}\right)^{-1} \exp\left[\Delta_{km}(k,l)\right]}^{Y_{km}(k,l)}}^{Y_k(k,l)}}{\underbrace{\sum_{i=1}^{N_{lvl}} \sum_{j=1}^{N_i} \underbrace{\left(\prod_{t=1}^{N_q} \sigma_{ij,t}\right)^{-1} \exp\left[\Delta_{ij}(k,l)\right]}_{Z_{ij}(k,l)}}_{Z(k,l)}} \qquad \text{(S3)}$$

Next, we define $Y_{km}(k,l)$ and $Z_{ij}(k,l)$ as the *km*th and *ij*th terms in the numerator and denominator sums. Finally, we define $Y_k(k,l) = \sum_{m=1}^{N_k} Y_{km}(k,l)$ and $Z(k,l) = \sum_{i=1}^{N_{lvl}} \sum_{j=1}^{N_i} Z_{ij}(k,l)$

as the numerator and denominator of the posterior probability, respectively. Dropping the (k,l) indexing on Y_k and Z for simplicity yields the following expression for the posterior probability

$$P\left(X_k \mid \mathbf{r}(k,l)\right) = \frac{Y_k}{Z}$$

The gradient of the log posterior probability for stimulus $\mathbf{s}_{kl}$ is therefore given by

$$\begin{aligned}\nabla_{\mathbf{f}_q} \log P\left(X_k \mid \mathbf{r}(k,l)\right) &= \nabla_{\mathbf{f}_q} \log Y_k - \nabla_{\mathbf{f}_q} \log Z \\ &= \frac{\nabla_{\mathbf{f}_q} Y_k}{Y_k} - \frac{\nabla_{\mathbf{f}_q} Z}{Z}\end{aligned} \quad \text{(S4)}$$

Expanding the numerator sum and distributing the gradient operator

$$\nabla_{\mathbf{f}_q} Y_k = \sum_{m=1}^{N_k} \nabla_{\mathbf{f}_q} Y_{km} \quad \text{(S5)}$$

Now, we evaluate $\nabla_{\mathbf{f}_q} Y_{km}$ with respect to $\mathbf{f}_q$. Note that the Z_{ij} terms in the denominator have the same form as the Y_{km} terms in the numerator (only the subscripts change). Therefore, from the gradient of the numerator, the gradient of the denominator follows directly with the relevant change in subscripts.

The gradient of Y_{km} is given by the product rule

$$\nabla_{\mathbf{f}_q} Y_{km} = \nabla_{\mathbf{f}_q}\left[\left(\prod_{t=1}^{N_q} \sigma_{km,t}^{-1}\right)\exp\left(\Delta_{km}\right)\right] \quad \text{(S6a)}$$

$$\nabla_{\mathbf{f}_q} Y_{km} = \left[\left(\prod_{t=1}^{N_q} \sigma_{km,t}^{-1}\right)\exp\left(\Delta_{km}\right)\underbrace{\nabla_{\mathbf{f}_q}\left[\Delta_{km}\right]}_{\text{equation A10}} + \exp\left(\Delta_{km}\right)\underbrace{\nabla_{\mathbf{f}_q}\left[\prod_{t=1}^{N_q} \sigma_{km,t}^{-1}\right]}_{\text{equation A11}}\right] \quad \text{(S6b)}$$

Thus, the gradient of Y_{km} depends on $\nabla_{\mathbf{f}_q}\left[\Delta_{km}\right]$ and $\nabla_{\mathbf{f}_q}\left[\prod_{t=1}^{N_q} \sigma_{km,t}^{-1}\right]$. First we evaluate the gradient of Δ_{km}. Then, we evaluate the gradient of $\prod_{t=1}^{N_q} \sigma_{km,t}^{-1}$. The gradient of Δ_{km} is

$$\nabla_{\mathbf{f}_q}\left[\Delta_{km}\right] = \nabla_{\mathbf{f}_q}\left(-\frac{1}{2}\sum_{t=1}^{N_q}\left(\frac{r_t(k,l) - r_t(k,m)}{\sigma_{km,t}}\right)^2\right) \quad \text{(S7)}$$

Applying the quotient rule

$$\nabla_{\mathbf{f}_q}\left[\Delta_{km}\right] = -\frac{1}{2}\frac{\sigma_{km,q}^2\left[\overbrace{\nabla_{\mathbf{f}_q}\left(r_{kl,q} - r_{km,q}\right)^2}^{\text{equation A9a}}\right] - \left(r_{kl,q} - r_{km,q}\right)^2\left[\overbrace{\nabla_{\mathbf{f}_q}\left(\sigma_{km,q}^2\right)}^{\text{equation A9b}}\right]}{\left(\sigma_{km,q}^2\right)^2} \quad \text{(S8)}$$

The two gradient terms in equation S8 evaluate to

$$\begin{aligned}\nabla_{\mathbf{f}_q}\left[\left(r_{kl,q}-r_{km,q}\right)^2\right]&=2\left(r_{kl,q}-r_{km,q}\right)\nabla_{\mathbf{f}_q}\left(r_{kl,q}-r_{km,q}\right)\\&=2\left(r_{kl,q}-r_{km,q}\right)\left(\mathbf{s}_{kl}-\mathbf{s}_{km}\right)\end{aligned}\tag{S9a}$$

$$\begin{aligned}\nabla_{\mathbf{f}_q}\sigma^2_{km,q}&=\nabla_{\mathbf{f}_q}\left(\alpha\left|r_{km,q}\right|+\sigma_0^2\right)\\&=\frac{r_{km,q}}{\left|r_{km,q}\right|}\alpha\mathbf{s}_{km}\end{aligned}\tag{S9b}$$

Plugging equations S9a and S9b into equation S8 and distributing terms yields an expression for the first gradient term in equation A6b. Specifically,

$$\nabla_{\mathbf{f}_q}\left[\Delta_{km}\right]=\left[-\frac{\left[\left(r_{kl,q}-r_{km,q}\right)\left(\mathbf{s}_{kl}-\mathbf{s}_{km}\right)\right]}{\left(\sigma^2_{km,q}\right)}\right]+\frac{1}{2}\frac{\left(r_{kl,q}-r_{km,q}\right)^2}{\left(\sigma^2_{km,q}\right)^2}\frac{r_{km,q}}{\left|r_{km,q}\right|}\alpha\mathbf{s}_{km}\tag{S10}$$

Now, we evaluate the second gradient term in equation A6b

$$\begin{aligned}\nabla_{\mathbf{f}_q}\left(\prod_{t=1}^{N_q}\sigma^{-1}_{km,t}\right)&=\nabla_{\mathbf{f}_q}\left(\sigma^{-1}_{km,q}\prod_{t\neq q}^{N_q}\sigma^{-1}_{km,t}\right)\\&=\left(\prod_{t\neq q}^{N_q}\sigma^{-1}_{km,t}\right)\left[\nabla_{\mathbf{f}_q}\left(\sigma^2_{km,q}\right)^{-1/2}\right]\\&=\left(\prod_{t\neq q}^{N_q}\sigma^{-1}_{km,t}\right)\left[-\frac{1}{2}\left(\sigma^2_{km,q}\right)^{-3/2}\nabla_{\mathbf{f}_q}\sigma^2_{km,q}\right]\\&=\left(\prod_{t\neq q}^{N_q}\sigma^{-1}_{km,t}\right)\left[-\frac{1}{2}\left(\sigma_{km,q}\right)^{-3}\nabla_{\mathbf{f}_q}\sigma^2_{km,q}\right]\\&=\left(\prod_{t=1}^{N_q}\sigma^{-1}_{km,t}\right)\left[-\frac{1}{2}\frac{\overbrace{\nabla_{\mathbf{f}_q}\sigma^2_{km,q}}^{\text{equation A9b}}}{\sigma^2_{km,q}}\right]\end{aligned}\tag{S11}$$

The expression for the last gradient term in equation S11 has already been determined (equation S9b). Also note that in all cases the gradient is taken with respect to each filter $\mathbf{f}_q$. Hence, the component of the gradient due to each filter $\mathbf{f}_q$ will only depend on the responses r_q and response standard deviations σ_q corresponding to that filter. Consequently, all other gradient components with a filter index not equal to $t \neq q$ will be zero.

Next, we substitute equation S10 and equation S11 into equation S6 and group terms to obtain a more compact expression for $\nabla_{\mathbf{f}_q}Y_{km}$

$$\nabla_{\mathbf{f}_q} Y_{km} = \left(\prod_{t=1}^{N_q} \sigma_{km,t}^{-1}\right) \exp\left(\Delta_{km}\right) \left[\overbrace{\nabla_{\mathbf{f}_q}\left[\Delta_{km}\right]}^{\text{equation A10}} - \frac{1}{2} \overbrace{\frac{\nabla_{\mathbf{f}_q} \sigma_{km,q}^{2}}{\sigma_{km,q}^{2}}}^{\text{equation A9b}} \right] \tag{S12}$$

Note that if the internal noise is assumed to be constant (i.e. fano factor set equal to zero) instead of scaled, the second bracketed term in S12 (labeled equation S9b) vanishes.

Now, all terms required for the gradient of the cost have been determined. The full expression for the gradient of the cost function is given by

$$\nabla_{\mathbf{f}_q} \bar{C} = -\frac{1}{N}\left[\sum_{kl}^{N} \frac{\nabla_{\mathbf{f}_q} Y_k}{Y_k} - \frac{\nabla_{\mathbf{f}_q} Z}{Z}\right] \tag{S13}$$
