# Supplementary material for: Accuracy Maximization Analysis for Sensory-Perceptual Tasks: Computational Improvements, Filter Robustness, and Coding Advantages for Scaled Additive Noise
Source: PLoS Comput Biol. 2017 Feb 8;13(2):e1005281. doi: 10.1371/journal.pcbi.1005281 (PMC5298250; doi:10.1371/journal.pcbi.1005281)
Supplement: S3 Text — (PDF) [file pcbi.1005281.s006.pdf]

## S3 Text: AMA gradient with the MSE cost function

Given the squared error cost function, the expected cost per stimulus can be written as

$$\bar{C}_{kl} = E_{\mathbf{R}(k,l)}\left[\left(\hat{X}_{kl}^{opt} - X_k\right)^2\right] \tag{S14}$$

For the squared error cost function, the optimal estimate is the mean of the posterior

$$\begin{aligned}\hat{X}_{kl}^{opt} &= E\left[X \mid \mathbf{R}(k,l)\right] \\ &= \sum_{u=1}^{N_{lvl}} X_u p\left(X_u \mid \mathbf{R}(k,l)\right)\end{aligned} \tag{S15}$$

Using the approximation that the expected cost of each stimulus is equal to the cost given the expected response and plugging S15 into S14, the cost for each stimulus is

$$\bar{C}_{kl} \cong \left[\left(\overbrace{\left[\sum_{u=1}^{N_{lvl}} X_u p\left(X_u \mid \mathbf{r}(k,l)\right)\right]}^{\hat{X}_{kl}^{opt}} - X_k\right)^2\right] \tag{S16}$$

The gradient of the cost each stimulus is

$$\begin{aligned}\nabla_{\mathbf{f}_q}\bar{C}_{kl} &= \nabla_{\mathbf{f}_q}\left(\hat{X}_{kl}^{opt} - X_k\right)^2 \\ &= 2\left(\hat{X}_{kl}^{opt} - X_k\right)\nabla_{\mathbf{f}_q}\hat{X}_{kl}^{opt}\end{aligned} \tag{S17}$$

The gradient of the optimal estimate given the mean response is

$$\nabla_{\mathbf{f}_q}\hat{X}_{kl}^{opt} = \sum_{u=1}^{N_{lvl}} X_u\left[\nabla_{\mathbf{f}_q} p\left(X_u \mid \mathbf{r}(k,l)\right)\right] \tag{S18}$$

The gradient of the posterior probability is related to the gradient of the log posterior probability by the expression

$$\nabla_{\mathbf{f}_q} p\left(X_u \mid \mathbf{r}(k,l)\right) = p\left(X_u \mid \mathbf{r}(k,l)\right)\overbrace{\nabla_{\mathbf{f}_q}\log p\left(X_u \mid \mathbf{r}(k,l)\right)}^{\text{equation A4}} \tag{S19}$$

(Note that equation S19 holds because $\nabla \log p(x) = \nabla p(x)/p(x)$ which is valid when $p(x)$ is always greater than zero, which it is in our case).

Substituting equation S4 into equation S19 with an appropriate change of subscript gives

$$\nabla_{\mathbf{f}_q} p\left(X_u \mid \mathbf{r}(k,l)\right) = \frac{Y_u}{Z}\left(\frac{\nabla_{\mathbf{f}_q} Y_u}{Y_u} - \frac{\nabla_{\mathbf{f}_q} Z}{Z}\right) \tag{S20}$$

The gradient of the optimal estimate for a given stimulus is obtained by substituting equation S20 into equation S18

$$\nabla_{\mathbf{f}_q} \hat{X}_{kl}^{opt} = \sum_{u=1}^{N_{lvl}} X_u \left[ \frac{Y_u}{Z} \left( \frac{\nabla_{\mathbf{f}_q} Y_u}{Y_u} - \frac{\nabla_{\mathbf{f}_q} Z}{Z} \right) \right] \tag{S21}$$

The full expression for the gradient of the MSE cost function is obtained by substituting equation S21 into equation S17, and then averaging the gradient of the cost over all stimuli. Namely,

$$\begin{aligned} \nabla_{\mathbf{f}_q} \bar{C} &= \frac{1}{N} \sum_{kl}^{N} \nabla_{\mathbf{f}_q} \bar{C}_{kl} \\ &= \frac{2}{N} \sum_{kl}^{N} \left( \hat{X}_{kl}^{opt} - X_k \right) \nabla_{\mathbf{f}_q} \hat{X}_{kl}^{opt} \end{aligned} \tag{S22}$$
