# Supplementary material for: Accuracy Maximization Analysis for Sensory-Perceptual Tasks: Computational Improvements, Filter Robustness, and Coding Advantages for Scaled Additive Noise
Source: PLoS Comput Biol. 2017 Feb 8;13(2):e1005281. doi: 10.1371/journal.pcbi.1005281 (PMC5298250; doi:10.1371/journal.pcbi.1005281)
Supplement: S4 Text — (PDF) [file pcbi.1005281.s007.pdf]

S4 Text: Optimal estimator for 0,1 cost function is MAP estimator
The cost function associated with the L0 norm is given by

$$\gamma\left(\hat{X},X\right)=E\left[\left(\hat{X}-X\right)^{0}\right]$$

$$\gamma\left(\hat{X},X\right)=\sum_{X}\left(\hat{X}-X\right)^{0}p(X)$$

Splitting the summation for all $\hat{X}$ that do and do not equal X

$$\gamma\left(\hat{X},X\right)=\sum_{X_k=\hat{X}}0\,p\left(X_k\right)+\sum_{\forall X\neq\hat{X}}p\left(X\right)$$

The first term disappears and the second term can be rewritten

$$\gamma\left(\hat{X},X\right)=1-p\left(X_k\right)$$

The value of $\hat{X}$ that corresponds to the maximally probable X minimizes the cost.

$$\hat{X}^{opt}=\arg\max_{X}p\left(X\right) \qquad \text{(S23)}$$

Thus, for the 0,1 cost function, the maximum a posteriori (MAP) estimator is the optimal estimator.
