# Supplementary material for: Accuracy Maximization Analysis for Sensory-Perceptual Tasks: Computational Improvements, Filter Robustness, and Coding Advantages for Scaled Additive Noise
Source: PLoS Comput Biol. 2017 Feb 8;13(2):e1005281. doi: 10.1371/journal.pcbi.1005281 (PMC5298250; doi:10.1371/journal.pcbi.1005281)
Supplement: S5 Text — (PDF) [file pcbi.1005281.s008.pdf]

S5 Text: KL-divergence is negative log-probability of correct latent variable
A measure of the difference between two probability distributions $p(X)$ and $p^*(X)$ is known as the Kullback-Leibler divergence

$$\begin{aligned} D &= \sum_X p^*(X) \log \frac{p^*(X)}{p(X)} \\ &= \sum_X p^*(X) \log p^*(X) - p^*(X) \log p(X) \end{aligned}$$

Assume that $p^*(X)$ is an idealized posterior probability distribution that has all of its mass at the correct value of the latent variable X_k; thus, $p^*(X \neq X_k) = 0$ and $p^*(X = X_k) = 1$. Partitioning the expression for $X \neq X_k$ and $X = X_k$

$$\begin{aligned} D = &\left[\sum_{X \neq X_k} p^*(X) \log p^*(X) - p^*(X) \log p(X) \right] + \cdots \\ &\left[p^*(X_k) \log p^*(X_k) - p^*(X_k) \log p(X_k) \right] \end{aligned}$$

Plugging in yields the expression for the KL-divergence used throughout the paper

$$D = -\log p(X_k) \tag{S24}$$

The KL-divergence cost (a slight abuse of terminology) for each stimulus is thus the negative log posterior probability $C_{kl} = -\log p(X_k \mid \mathbf{R}(k,l))$. The 0,1 cost is the negative posterior probability $C_{kl} = 1 - p(X_k \mid \mathbf{R}(k,l))$ at the correct value of the latent variable (see S4 Text).
