# Supplementary material for: Accuracy Maximization Analysis for Sensory-Perceptual Tasks: Computational Improvements, Filter Robustness, and Coding Advantages for Scaled Additive Noise
Source: PLoS Comput Biol. 2017 Feb 8;13(2):e1005281. doi: 10.1371/journal.pcbi.1005281 (PMC5298250; doi:10.1371/journal.pcbi.1005281)
Supplement: S6 Text — (PDF) [file pcbi.1005281.s009.pdf]

S6 Text: Posterior mean is optimal estimator for squared error cost function

The cost function associated with the L2 norm is given by

$$\gamma\left(\hat{X},X\right)=E\left[\left(\hat{X}-X\right)^2\right]$$

$$\gamma\left(\hat{X},X\right)=\sum_X\left(\hat{X}-X\right)^2 p\left(X\right)$$

To find its minimum, first differentiate with respect to $\hat{X}$ …

$$\frac{\partial\gamma\left(\hat{X},X\right)}{\partial\hat{X}}=2\hat{X}\sum_X p\left(X\right)-2\sum_X Xp\left(X\right)$$

The optimum estimator is the value of $\hat{X}$ that sets the derivative to zero.

$$0=2\hat{X}^{opt}\sum_X p\left(X\right)-2\sum_X Xp\left(X\right)$$

The first summation is equal to 1, so we have

$$0=2\hat{X}^{opt}-2\sum_X Xp\left(X\right)$$

Solving for $\hat{X}^{opt}$

$$\hat{X}^{opt}=\sum_X Xp\left(X\right)$$

The right hand side of the equation is the definition of expectation (i.e. the posterior mean)

$$\hat{X}^{opt}=E\left[X\right] \qquad \text{(S25)}$$
