# Supplementary material for: Accuracy Maximization Analysis for Sensory-Perceptual Tasks: Computational Improvements, Filter Robustness, and Coding Advantages for Scaled Additive Noise
Source: PLoS Comput Biol. 2017 Feb 8;13(2):e1005281. doi: 10.1371/journal.pcbi.1005281 (PMC5298250; doi:10.1371/journal.pcbi.1005281)
Supplement: S7 Text — (PDF) [file pcbi.1005281.s010.pdf]

S7 Text: Rotating correlated filters within the spanned subspace
Here, we derive the expressions for the weighted linear combinations that rotate two, arbitrary vectors within the subspace that they span. These linear combinations preserve filter length and correlation (i.e. cosine similarity)

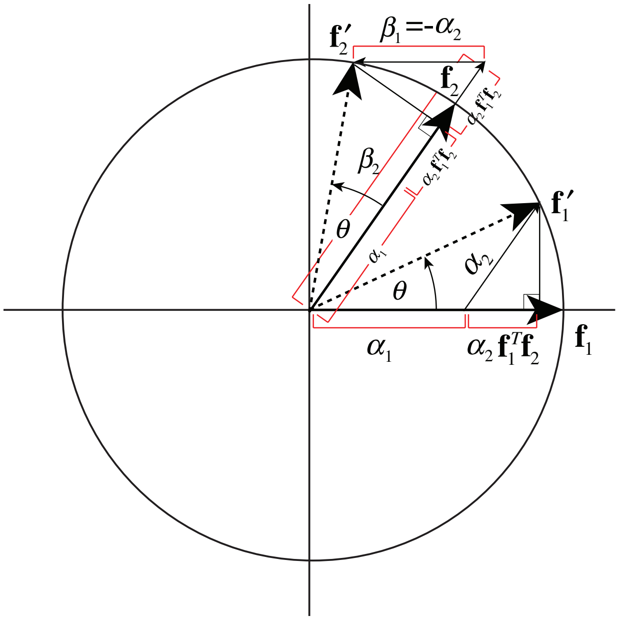

Any two N-dimensional unit vectors, $\mathbf{f}_1$ and $\mathbf{f}_2$, define a two-dimensional subspace in $\mathbb{R}^N$ assuming that $\mathbf{f}_2 \neq \pm\mathbf{f}_1$. The weighted linear combination that rotates the vectors by angle θ while preserving length and filter correlation $\rho = \mathbf{f}_2^T\mathbf{f}_1 = \mathbf{f}_2'^T\mathbf{f}_1'$ is given by

$$\mathbf{f}_1' = \alpha_1\mathbf{f}_1 + \alpha_2\mathbf{f}_2 \quad \text{(S26a)}$$

$$\mathbf{f}_2' = \beta_1\mathbf{f}_1 + \beta_2\mathbf{f}_2 \quad \text{(S26b)}$$

To determine the weights, consider the triangle formed by the origin, $\mathbf{f}_1$ and $\mathbf{f}_1'$. By assumption, the hypotenuse has length 1.0. The rotation angle θ is related to weights α_1 and α_2 by the trigonometric equations relating sin(.) and cos(.) to the opposite and adjacent sides of the unit right triangle

$$\sin\theta = \sqrt{\alpha_2^2 - \alpha_2^2\rho^2} \quad \text{(S27a)}$$

$$\cos\theta = \alpha_1 + \alpha_2\rho \quad \text{(S27b)}$$

Rearranging equation S27a yields the expression for α_2 in terms of θ and ρ

$$\alpha_2 = \frac{\sin\theta}{\sqrt{1-\rho^2}} \quad \text{(S28)}$$

Plugging equation S28 into equation S27b expresses α_1 in terms of θ and ρ

$$\alpha_1 = \cos\theta - \frac{\sin\theta}{\sqrt{1-\rho^2}}\rho \quad \text{(S29)}$$

The weights β can be expressed in terms of the weights α by observing the similarity of the triangles formed by the origin, $\mathbf{f}_1$, and $\mathbf{f}_1'$, and the origin, $\mathbf{f}_2$, and $\mathbf{f}_2'$

$$\beta_1 = -\alpha_2 \tag{S30a}$$

$$\beta_2 = \alpha_1 + 2\alpha_2\rho \tag{S30b}$$

Representing in matrix form

$$\begin{bmatrix} | & | \\ \mathbf{f}_1' & \mathbf{f}_2' \\ | & | \end{bmatrix} = \begin{bmatrix} | & | \\ \mathbf{f}_1 & \mathbf{f}_2 \\ | & | \end{bmatrix} \begin{bmatrix} \alpha_1 & -\alpha_2 \\ \alpha_2 & \alpha_1 + 2\alpha_2\rho \end{bmatrix} \tag{S31}$$

Equations S28-31 specify the length and angle preserving linear transformations that rotate an arbitrary pair of unit vectors by angle θ within the subspace that they span.
