# Supplementary material for: Accuracy Maximization Analysis for Sensory-Perceptual Tasks: Computational Improvements, Filter Robustness, and Coding Advantages for Scaled Additive Noise
Source: PLoS Comput Biol. 2017 Feb 8;13(2):e1005281. doi: 10.1371/journal.pcbi.1005281 (PMC5298250; doi:10.1371/journal.pcbi.1005281)
Supplement: S8 Text — (PDF) [file pcbi.1005281.s011.pdf]

S8 Text: Uncertainty ellipses for encoding with correlated filters in standard basis
Here, we seek the uncertainty ellipse in the standard basis associated with the noisy encoding of a stimulus with two arbitrary possibly correlated filters having independent noise. We start by finding the linear mapping $\mathbf{f} \rightarrow \mathbf{e}$ where $\mathbf{e}$ is an orthonormal basis spanning the same subspace as $\mathbf{f}$. Specifically,

$$\mathbf{e}_1 = \alpha_1 \mathbf{f}_1 + \alpha_2 \mathbf{f}_2 \qquad \text{(S32a)}$$

$$\mathbf{e}_2 = \beta_1 \mathbf{f}_1 + \beta_2 \mathbf{f}_2 \qquad \text{(S32b)}$$

such that $\mathbf{e}_1 = \mathbf{f}_1$ and $\mathbf{e}_2^T \mathbf{e}_1 = 0$, and where $\rho = \mathbf{f}_2^T \mathbf{f}_1$. The weights are given by

$$\alpha_1 = 1 \quad ; \quad \alpha_2 = 0$$

$$\beta_1 = -\rho \big/ \sqrt{1-\rho^2} \quad ; \quad \beta_2 = 1 \big/ \sqrt{1-\rho^2}$$

which can be seen directly from the diagram below:

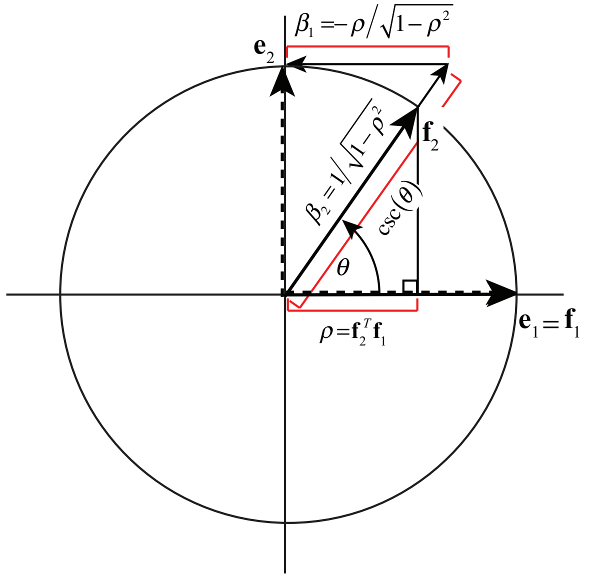

The linear mapping in matrix form is

$$\begin{bmatrix} | & | \\ \mathbf{e}_1 & \mathbf{e}_2 \\ | & | \end{bmatrix} = \begin{bmatrix} | & | \\ \mathbf{f}_1 & \mathbf{f}_2 \\ | & | \end{bmatrix} A \qquad \text{(S33)}$$

where $A = \begin{bmatrix} 1 & -\rho \big/ \sqrt{1-\rho^2} \\ 0 & 1 \big/ \sqrt{1-\rho^2} \end{bmatrix}$

The inverse mapping is obtained by right-multiplying both sides of S33 with A^{-1}. The matrix can also be used to convert the coordinates of the stimulus projection in the standard basis (see below) to the filter responses.

The coordinates and the covariance of the uncertainty ellipse in the standard basis are

$$\mathbf{r}^{\{std\}} = A^T \mathbf{r} = \mathbf{e}^T \mathbf{s} \tag{S34}$$

$$\Sigma^{\{std\}} = A^T \Sigma A \tag{S35}$$

where $\mathbf{r}$ and Σ are the joint response mean and noise covariance matrix in the filter basis, and $\mathbf{r}^{\{std\}} = \mathbf{e}^T \mathbf{s}$ and $\Sigma^{\{std\}}$ are the coordinates and covariance of the uncertainty ellipse in the standard basis.

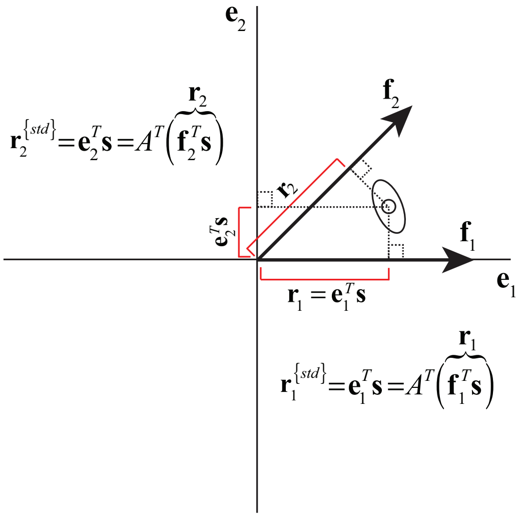

Expanding the matrix multiplication

$$\mathbf{r}^{\{std\}} = \begin{bmatrix} \mathbf{r}_1 \\ (\mathbf{r}_2 - \mathbf{r}_1\rho)/\sqrt{1-\rho^2} \end{bmatrix} \tag{S36}$$

$$\Sigma^{\{std\}} = \begin{bmatrix} \sigma_1^2 & -\sigma_1^2\rho/\sqrt{1-\rho^2} \\ -\sigma_1^2\rho/\sqrt{1-\rho^2} & (\sigma_1^2\rho^2 + \sigma_2^2)/(1-\rho^2) \end{bmatrix} \tag{S37}$$

The smaller and larger eigenvalues of this covariance of the encoding

$$\lambda_1^{\{std\}} = \frac{1}{2(1-\rho^2)}\left[\sigma_1^2 + \sigma_2^2 - \sqrt{(\sigma_1^2 - \sigma_2^2)^2 + 4\sigma_1^2\sigma_2^2\rho^2}\right] \tag{S38a}$$

$$\lambda_2^{\{std\}} = \frac{1}{2(1-\rho^2)}\left[\sigma_1^2 + \sigma_2^2 + \sqrt{(\sigma_1^2 - \sigma_2^2)^2 + 4\sigma_1^2\sigma_2^2\rho^2}\right] \tag{S38b}$$

Interestingly, in the limit as $\rho \to 1$, the larger eigenvalue goes to infinity, and the smaller eigenvalue reduces (via L'Hospital's rule) to the familiar expression for the variance of the optimal linear combination of conditionally independent Gaussian random variables

$$\lim_{\rho \to 1} \lambda_1^{\{std\}} = \frac{\sigma_1^2\sigma_2^2}{\sigma_1^2 + \sigma_2^2} \tag{S39a}$$

$$\lim_{\rho \to 1} \lambda_2^{\{std\}} = \infty \tag{S39b}$$
